# Removing ballistocardiogram (BCG) artifact from full-scalp EEG acquired inside the MR scanner with Orthogonal Matching Pursuit (OMP)

**DOI:** 10.3389/fnins.2014.00218

**Published:** 2014-07-29

**Authors:** Hongjing Xia, Dan Ruan, Mark S. Cohen

**Affiliations:** ^1^Department of Bioengineering, University of CaliforniaLos Angeles, CA, USA; ^2^Department of Radiation Oncology, University of CaliforniaLos Angeles, CA, USA; ^3^Department of Psychiatry, Neurology, Radiology, Psychology, Biomedical Physics, California NanosSystems Institute, University of CaliforniaLos Angeles, CA, USA

**Keywords:** ballistocardiogram, simultaneous EEG-fMRI, artifact removal, orthogonal matching pursuit (OMP), subset selection, signal separation, brain rhythms, alpha waves

## Abstract

Ballistocardiogram (BCG) artifact remains a major challenge that renders electroencephalographic (EEG) signals hard to interpret in simultaneous EEG and functional MRI (fMRI) data acquisition. Here, we propose an integrated learning and inference approach that takes advantage of a commercial high-density EEG cap, to estimate the BCG contribution in noisy EEG recordings from inside the MR scanner. To estimate reliably the full-scalp BCG artifacts, a near-optimal subset (20 out of 256) of channels first was identified using a modified recording setup. In subsequent recordings inside the MR scanner, BCG-only signal from this subset of channels was used to generate continuous estimates of the full-scalp BCG artifacts via inference, from which the intended EEG signal was recovered. The reconstruction of the EEG was performed with both a direct subtraction and an optimization scheme. We evaluated the performance on both synthetic and real contaminated recordings, and compared it to the benchmark Optimal Basis Set (OBS) method. In the challenging non-event-related-potential (non-ERP) EEG studies, our reconstruction can yield more than fourteen-fold improvement in reducing the normalized RMS error of EEG signals, compared to OBS.

## 1. Introduction

Simultaneous electroencephalography and functional magnetic resonance imaging acquisition offers a promising probe to study different, yet connected, bioelectric and hemodynamic attributes of brain activity with complementary temporal and spatial resolutions. This non-invasive neuroimaging technique has applications in the analysis of event-related brain responses (Eichele et al., 2005; Debener et al., 2006; Benar et al., 2007), studies of ongoing brain rhythms and networks (Goldman et al., 2002; Laufs et al., 2003), and studies of epileptic activity (Krakow et al., 2001; Lemieux et al., 2001; Bénar et al., 2003). Despite many successful applications, ballistocardiogram (BCG) artifact in concurrent EEG-fMRI acquisition still presents a challenge in continuous recoding (e.g., non-ERP) studies especially when the magnetic field strength is high. BCG presents high temporal non-stationarity due to variation in cardiac cycles (Bonmassar et al., 2002; Debener et al., 2007), and its amplitude scales with magnetic field strength (Yan et al., 2010; Mullinger et al., 2013).

Previous attempts to suppress the BCG artifacts have focused primarily on channel-wise denoising, with major developments in template-subtraction, principal component analysis (PCA)-based methods (Allen et al., 2000; Goldman et al., 2000; Ellingson et al., 2004; Niazy et al., 2005) and independent component analysis (ICA)-based methods (Srivastava et al., 2005; Ghaderi et al., 2010; Liu et al., 2012), as reviewed in Grouiller et al. (2007) and Vanderperren et al. (2010). The widely used Optimal Basis Sets method (OBS) (Niazy et al., 2005) is a PCA-based approach that regresses out the mean effects and its first few principal components from the contaminated data on a heartbeat-by-heartbeat basis. Attempts to incorporate spatial information have also been made with spatial PCA and ICA by Bénar et al. (2003) and Srivastava et al. (2005). However these PCA/ICA-based approaches are based on strong orthogonality/independence assumptions and subject to manual selection of number of components to be included.

Another focus on BCG suppression is based on reference signals, generated by motion sensors (Bonmassar et al., 2002) or wire loops (Masterton et al., 2007), for the artifact itself. More recent developments, such as Chowdhury et al. (2014), Mullinger et al. (2013) and Xia et al. (2013b) and the fEEG^TM^ system from (Kappametrics Inc., Chantilly, USA), apply an insulating layer to directly acquire BCG-only artifact signals from channels that are electrically isolated from the scalp. Although the measured artifact reference signals are not identical to the BCG (Mullinger et al., 2013), significant suppression has been achieved by reference layer artifact subtraction (RLAS) (Chowdhury et al., 2014). However, RLAS and fEEG^TM^ require purpose-built hardware and exploit no further denoising steps than a simple subtraction.

We propose a method to remove BCG from the uninsulated channels using inferential relationship amongst whole-scalp BCG signals, which provides an additional denoising benefit yet requires no hardware modification. As the BCG artifacts (Yan et al., 2010; Mullinger et al., 2013) are related to the movements of conductive liquid such as surface blood flow, or movements from electrodes caused by pulsation of blood vessels or head motion, we expect similar BCG temporal behaviors from adjacent channels. In our previous study Xia et al. (2014), we proposed surrounding each uninsulated channel with a neighborhood of shielded channels that provide BCG-only signals, to ensure access to at least one proper prior. This approach, though performs well, is limited by its *ad hoc* neighboring channel selection and the potential requirement of a large number of insulated channels. Therefore, a sparse, and stable, insulation pattern is highly desirable in contrast to local probing in order to explore brain activity patterns.

We aim here to balance two conflicting goals: (1) minimize the number of insulated channels; and (2) denoise the EEG signals in the uninsulated channels with high accuracy. In an optimization framework, we jointly seek the optimal subset of a small cardinality to insulate, and an inference model to estimate the BCG components for the other uninsulated channels based on the BCG readings from the insulated set. We propose here a simple greedy scheme based on orthogonal matching pursuit (OMP), and report its performance in comparison with both the benchmark OBS method and inference with two alternative *ad hoc* insulation patterns.

## 2. Generative model for contaminated EEG data

As BCG and EEG are believed to originate from independent sources, they should add linearly with minimal interaction and subject to noise contamination. Mathematically,

(1) $$Y=X_{bcg}+X_{eeg}+\varepsilon ,$$

where **X**_*bcg*_, **X**_*eeg*_ and ε ∈ ℝ^*C× T*^ represent BCG artifacts, underlying uncontaminated EEG signals, and noise respectively. *C* and *T* are the number of channels and the number of time points of the recordings, respectively. This model does not presume any statistical relationship between BCG and EEG: independence of the noise sources is in the sense of physics and physiology, rather than statistics. This model has been applied implicitly in many previous studies and simulation studies Allen et al. (2000), Goldman et al. (2000), Niazy et al. (2005), Grouiller et al. (2007) and Vanderperren et al. (2010).

## 3. Experimental setup

Three healthy right-handed adult volunteers, (2 male and 1 female, with age between 24 and 28 years), gave informed consent for participation in this study according to the guidelines of the UCLA medical investigational review board. For our experiments, we used a 3T Siemens Tim Trio scanner (Siemens Medical Solutions, Erlangen, Germany). We acquired EEG data with a GES300MR system (Electrical Geodesics, Inc., Eugene OR). This 256-channel apparatus made contact with the scalp via KCL-filled sponge contacts mounted in plastic pedestals with a contact-impedance of 20 kΩ or less. EEG data were sampled at 250 Hz and amplifier gains were kept constant. To focus on only BCG artifacts, no MRI scanning took place during the acquisitions inside the scanner. The overall protocol is kept consistent for recording spontaneous EEG as well as eyes open/close EEG activity (see more experimental setup details in Xia et al., 2014).

### 3.1. Acquisition of BCG-only data

**Figure 1:**
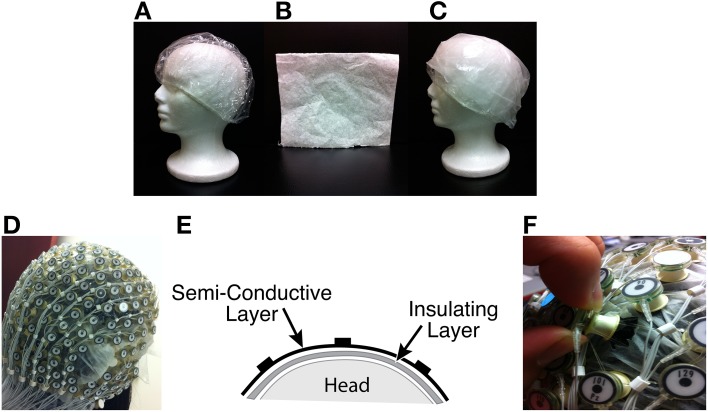
**(A)** Insulation layer: a shower cap **(B)** Semi-conducting layer: paper layer **(C)** A piece of thin paper dampened with saline placed on top of the insulation layer **(D)** A picture with all channels blocked **(E)** Sandwich diagram of construction **(F)** Unblocking one channel.

Two layers of material were inserted between the scalp and the electrodes to collect BCG-only data while electrically blocking conductance of EEG brain signals, as shown in Figures 1C–E.

1. Insulating Layer: To collect BCG-only artifacts, we first isolated electrodes from the scalp with a plastic insulating barrier to block brain signals from conduction, as shown in Figure 1A.
2. Semi-conducting Layer: To collect properly signals from insulated electrodes, a semi-conductive layer was then inserted between the insulating layer and the electrodes. For this we used a thin piece of paper, dampened with saline (Figure 1B), as the semi-conductive layer, which provided the proper impedance while avoiding short circuits or alteration of BCG signals.

### 3.2. Acquisition of contaminated EEG and BCG-only data

Inside the scanner, we acquired contaminated and BCG-only data at the same time, but from different channels. After inserting two layers for acquiring BCG-only signals, we recorded simultaneously contaminated EEG data from selected channels by removing the insulation and paper layers underneath, as shown in Figure 1F. We chose to unblock 20 conventional channels, approximating the standard 10–20 systems. In practice, and as discussed below, one can determine which channels to block in advance, and use setup in Figure 1F to maximize the number of channels that collect EEG signals. The measured impedance before and after unblocking and their difference are provided in the Supplementary Material. On average, the impedance differed by 100Ω for electrodes in the blocked, and unblocked, conditions.

## 4. General inference logic and work flow

**Figure 2:**
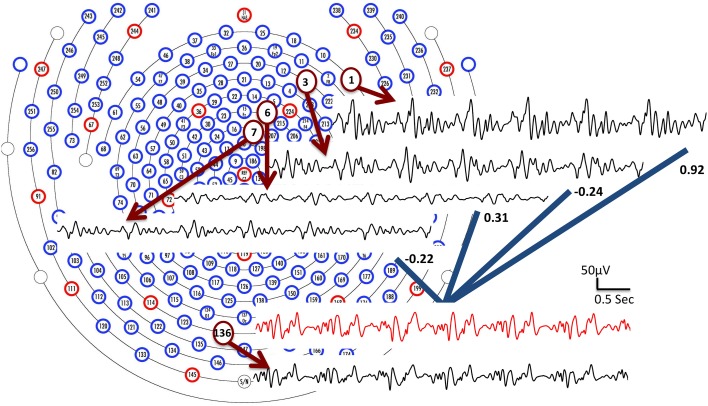
**BCG traces from four channels (1, 3, 6, and 7) are combined linearly with weights to form an estimate of the BCG trace from channel 136 (shown in red)**. The collected BCG signals (channel 1, 3, 6, 7 and 136) are shown in black.

BCG signals, which are linked to pulsation and other motion effects (Niazy et al., 2005; Mullinger et al., 2013), exhibit high temporal non-stationarity, making direct temporal modeling extremely difficult with classic parametric/nonparametric approaches. Despite this, we expect the correlations of the BCG traces across multiple channels to be approximately consistent, as illustrated in Figure 2. We hypothesize that the full-scalp BCG-only signals can be of intrinsically low dimension along the spatial direction when signals acquired from multiple locations contain redundant/correlated information; this is supported by the observation (see the Supplementary Material) that just four principal components (PCs) explain more than 95% total energy after applying spatial PCA to the full-scalp BCG-only signals, **X**_*bcg*_ ∈ ℝ^*C× T*^. A similar observation was made also in Bénar et al. (2003) via visual inspection. These preliminary analyses allude to the possibility of inferring the full-scalp BCG artifacts from BCG signals collected from a subset of channels.

In the equations below, we adopt several MATLAB (the Mathworks, Natick, MA) notations for their compactness and clarity. For any subset Λ ⊆ {1,2,…,*C*} and matrix **X**∈ ℝ^*C× T*^, **X**[Λ,:] denotes a submatrix of **X** consisting of rows **X**_*i*_ for *i* ∈ Λ. The cardinality of the set Λ is denoted by |Λ|. In this paper, we describe a set of full-scalp channels as Λ_*full*_ (|Λ_*full*_| = *C*), the subset of insulated channels as Λ_*ins*_, and the complementary set of non-insulated channels as Λ_*nins*_ = Λ_*full*_\Λ_*ins*_. The linear inference seeks a relation with

(2) $$X_{bcg}[\Lambda_{nins},:]=WX_{bcg}[\Lambda_{ins},:]+\text{noise},$$

where **W** is of dimension |Λ_*nins*_| × |Λ_*ins*_|. We propose a two-stage procedure to estimate the full-scalp BCG artifacts with BCG-only signals from a subset of insulated channels, based on the assumption that the spatial correlation of full-scalp BCG is relatively consistent. See Section 4.4 and 5.2.1 for greater detail.

In the first stage for model building, all channels are insulated to collect full-scalp BCG-only signals, **X**_*bcg*_, which are used to estimate an inference matrix **W** and select a subset Λ_*ins*_ via Equation 2. In the second stage for acquisition, the channels in Λ_*ins*_ remain insulated to acquire BCG-only signals **X**_*bcg*_[Λ_*ins*_,:] while the remainder of the channels Λ_*nins*_ collects normal contaminated EEG recordings **Y**[Λ_*nins*_,:] in which the BCG contributions are estimated subsequently via Equation 2 with the inference, **W**, from the model-building stage.

### 4.1. Stage I: model building

It is desirable to use a small number of channels for BCG estimations, so that the proposed approach can be applied to a wide range of EEG caps and preserve the value of high-density EEG recordings. We choose a “budget” size (the cardinality of insulation set |Λ_*ins*_|) to be 20 by cross validation, as reported in section 5.2.2. The goal of selecting the optimal subset of electrodes with the best inference performance can be formulated into a minimization-minimization problem:

(3) $$\underset{\Lambda_{ins}}{\min}\{\underset{W(\Lambda_{ins})}{\min}{\left\|X_{bcg}[\Lambda_{nins},:]-WX_{bcg}[\Lambda_{ins},:]\right\|}_{F}^{2}\},$$

where ‖‖_*F*_ denotes Frobenius norm and we use **W**(Λ_*ins*_)to explicitly indicate the dependence of the optimal **W** on the subset Λ_*ins*_. By introducing an identity map on the insulated portion and reformulation with an expanded inference matrix $\tilde{W}$ = [**I**;**W**], it can be shown that optimizing Equation 3 is equivalent to solving

(4) $$\underset{\Lambda_{ins}}{\min}\{\underset{\tilde{W}}{\min}{\left\|X_{bcg}[\Lambda_{full},:]-\tilde{W}X_{bcg}[\Lambda_{ins},:]\right\|}_{F}^{2}\},$$

which has a regression goal independent of the insulation set Λ_*ins*_. The inner problem of solving for $\tilde{W}$ given Λ_*ins*_ is an ℓ_2_ problem with a closed-form solution, but the outer set selection problem is NP-hard. For practical purposes, we adopt a greedy Orthogonal Matching Pursuit (OMP) approach (Tropp and Gilbert, 2007) for the set selection problem. At each step the subset Λ_*ins*_ is expanded by one channel that maximizes the inner product of the signal from the selected channel and the residual signals not yet explained by the already selected channels. The inference matrix, $\tilde{W}$, is updated at each step with an updated subset Λ_*ins*_.

The OMP procedure is as follows:

- Step 1: Initialize the insulating subset as empty Λ^(0)^_*ins*_ = {}, the inference matrix $\tilde{W}$^(0)^ = **0**, and the residual signals **R**^(0)^ = **X**_*bcg*_[Λ_*full*_,:]. Then, initialize the full-scalp BCG-only signals to be **X** = **X**_*bcg*_[Λ_*full*_,:], and set the iteration counter *k* = 1.
- Step 2: Find a channel that solves the maximization problem:
  $$i=\arg\underset{i}{\max}{\sum_{j\in \Lambda_{full}}\langle R^{\left(k-1\right)}{}_{j}\;,\frac{X_{i}}{{\left\|X_{i}\right\|}_{F}}\rangle }^{2}.$$
- Step 3: With this selection, update everything as follows:
  $$\begin{array}{l} \Lambda^{\left(k\right)}{}_{ins}=\Lambda^{\left(k-1\right)}{}_{ins}\;,\cup \left\{i\right\};\\ \;{\tilde{W}}^{\left(k\right)}=\arg\underset{\tilde{W}}{\min}{\left\|X_{bcg}[\Lambda_{full},:]-\tilde{W}X_{bcg}[\Lambda^{\left(k\right)}{}_{ins},:]\right\|}_{F}^{2};\\ \;R^{\left(k\right)}=X_{bcg}[\Lambda_{full},:]-{\tilde{W}}^{\left(k\right)}X_{bcg}[\Lambda_{ins}{}^{\left(k\right)},:];\\ \text{ k=k+1.} \end{array}$$
  Go back to step 2 until the budget number of insulated channels has been reached.
- Step 4: Store the final set Λ_*ins*_ = Λ^(*k*)^_*ins*_ and the inference matrix $\tilde{W}$ = $\tilde{W}$^(*k*)^.

After obtaining the inference matrix $\tilde{W}$ and insulation subset Λ_*ins*_ from the full-scalp BCG-only signals, the estimated BCG components $\hat{X}$_*bcg*_[Λ_*nins*_,:] in the contaminated EEG recordings are reconstructed according to Equation 2 using simultaneously collected BCG-only signals **X**_*bcg*_[Λ_*ins*_,:] by the following solution:

(5) $${\hat{X}}_{bcg}[\Lambda_{nins},:]=WX_{bcg}[\Lambda_{ins},:].$$

**Figure 3:**
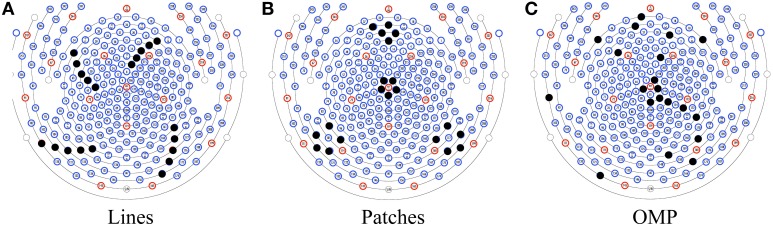
**Three patterns for insulating channels (solid black dots): (A) the Lines pattern, (B) the Patches pattern, and (C) a pattern selected by OMP**.

As an alternative to automatic set selection methods, we devised two *ad hoc* patterns: a “lines” pattern with 4 groups of 5 channels, as shown in Figure 3A; and a “patches” pattern containing 4 groups of 5 channels arranged in circles, as shown in Figure 3B. In addition, the selected pattern from OMP is presented in Figure 3C.

### 4.2. Stage II: reconstruction of clean EEG

Here, we investigate two methods for reconstruction: direct subtraction approach, and optimization-based.

1. Direct subtraction: A straightforward and assumption-free method for denoising EEG is to subtract the estimated BCG components directly from the recorded noisy data **Y**:
  (6) $${\hat{X}}_{eeg}[\Lambda_{nins},:]=Y[\Lambda_{nins},:]-{\hat{X}}_{bcg}[\Lambda_{nins},:].$$
2. Optimization-based reconstruction: To further separate EEG from BCG, we incorporate an optimization-based scheme for temporally-concatenated segments that utilizes prior information from the EEG acquired outside the scanner. Based on patterns observed from BCG-only and EEG-only data, we devised a regularized optimization framework to separate the signals using group sparsity technique developed in compressive sensing (Deng et al., 2011). The detailed explanation is provided in our previous work Xia et al. (2014). Mathematically, we impose a group sparsity penalty with ℓ_2,1_ norm, ${\left\|C_{eeg}\right\|}_{2,1}\overset{def}{=}\sum_{i=1}^{m}\|C_{eeg}[i,:]{\|}_{2}$, on the reconstructed EEG coefficient **C**_*eeg*_, where *i* ∈ {1,…,*m*} is an index set indicating the *i^th^* group (row), and *m* is the number of rows in **C**_*eeg*_. In addition, the energy function adopts the EEG basis **B**_*e_prior*_ learned from the out-of-scanner experiment. Therefore, one may reconstruct the EEG signals by estimating the EEG coefficients, **C**_*eeg*_, corresponding to the learned basis, **B**_*e_prior*_ for each uninsulated channel by minimizing:
  (7) $$\underset{C_{eeg}}{\min}{\left\|C_{eeg}\right\|}_{2,1}+\frac{1}{2}\mu {\left\|Y_{eeg}-B_{e\_prior}C_{eeg}\right\|}_{F}^{2},$$
  where the first term regularizes the group sparsity structure of the coefficients, and the second term imposes data fidelity. The scalar parameter, μ, balances the contribution of these two terms. **Y**_*eeg*_ is derived by subtracting the estimated BCG from recorded noisy data from one channel. The EEG component of the uninsulated channel is recovered by multiplying the prior basis with the estimated coefficients.

### 4.3. Construction of synthetic contaminated EEG data

To compare the EEG reconstruction performance quantitatively among different artifact removal methods, we simulate contaminated EEG data by combining 12 min of BCG-only, and EEG signals (Equation 1) (see more details in Xia et al., 2013b). This provides us with access to ground-truth that is absent in normal EEG-fMRI acquisitions. We use “clean” EEG signals collected from outside the scanner. Our simulation differs from the published works (Grouiller et al., 2007; Ghaderi et al., 2010; Vanderperren et al., 2010), in that we use the true BCG signals to synthesize the contaminated data. The inference models used in the comparison include two *ad hoc* patterns, “lines” and “patches,” and the pattern from the OMP approach. We compare the performance among three methods: Channel-wise OBS (EEGLAB plug-in FMRIB version 1.2 (Niazy et al., 2005) with 3 principal components), inference + direct subtraction, and inference + optimization.

### 4.4. Consistency of the inference relationship

Our method assumes substantial consistency of the inference relationship over time, which reflects the temporal consistency of BCG spatial correlations among multiple channels. Once an inference matrix is sufficient in estimating one BCG segment, it is assumed to be adequate in recovering other segments that are distant in time. We perform a validation test with a 13-min full-scalp BCG-only recordings in section 5.2.1.

A second assumption of our methods is of nominal consistency of the inference relationship across experimental sessions. Between stage I and II sessions, subjects are removed from the scanner, and their caps are replaced. As a result of the physical movements, artifacts may differ substantially due to variations of scalp-electrode impedances and channel locations. Two kinds of inconsistencies may occur regarding our inference model. First, the channels selected from the first session may not suffice to represent BCG from another. Second, even if the selected channels remain representative, the inference matrix from the first session may not carry the proper weights to reconstruct the BCG from the second.

To verify the representativeness of selected channels, the subset selected from one training session is used to recover BCG from a testing session with an optimal inference matrix calculated from also the testing session, minimizing the contribution of the inconsistent inference matrix. After demonstrating the representativeness of selected channels, we proceed to examine the impact of physical movements on inference matrix. The inference matrix learned from the training session is applied to recover BCG from other testing sessions. The mean reconstruction errors of the two inconsistency tests are presented in Tables 1, **3**.

**Table 1:** **Statistical results of one occipital channel from 3 subjects**.

	**Subject 1**			**Subject 2**			**Subject 3**		
	**EC**	**EO**	***p*-value**	**EC**	**EO**	***p*-value**	**EC**	**EO**	***p*-value**
Contaminated	341.6	330.8	1.8e-01	1781.9	1790.3	6.6e-01	695.2	676.4	3.8e-01
OBS	74.1	59.6	2.1e-02	75.9	65.0	4.5e-02	72.3	56.2	3.1e-03
OMP+Sub	26.7	15.4	5.1e-06	8.2	3.1	9.1e-07	5.4	1.1	1.3e-25
OMP+Opt	26.6	15.3	4.4e-06	8.1	3.0	7.1e-08	5.2	0.9	6.5e-26

*EC columns contain mean (μV^2^) alpha power when the subjects eyes were closed. EO columns contain mean (μV^2^) alpha power when the eyes were open. *P*-values are from the Wilcoxon test. No significant change in alpha power was detected in the contaminated signal, while the OMP-based and OBS methods display the expected decreases from EC to EO conditions*.

In addition, we propose an inference matrix recalculation method to be used in the event that the inference matrix varies too much to recover adequate BCG. Our method divides time series signals for each channel into segments according to a fixed number of heartbeats, then computes the averages of the segments for each channel. As noted by others (Allen et al., 2000; Niazy et al., 2005), it is safe to assume that such ECG-synced averages contains negligible EEG after applying a 1-Hz high-pass filter to remove slow drifts in EEG, and only averaged BCG segments. As a result, we can obtain a new inference matrix from the BCG-only segments of all channels, and recover BCG following the same steps as section 4. This amendment operates on the temporal domain of full-scalp BCG signals with negligible alteration of the BCG spatial relationships among channels. The BCG reconstruction errors from the new matrix are theoretically (see the Supplementary Material) and experimentally (Section 5.2.1) proven to be small. Furthermore, we applied the consistency tests not only on 5-min BCG-only recordings from different experimental sessions from one subject, but also on 9-min BCG-only recordings from three subjects. The results are shown in section 5.2.1.

## 5. Results

When ground truth **X**_*i*_ is available, we define the normalized root mean squared error *nRMSE_i_* = ‖**X**_*i*_ − $\hat{X}$_*i*_‖_2_/‖**X**_*i*_‖_2_ for the channel index *i* to quantify the performance of either BCG or EEG estimation $\hat{X}$_*i*_. This channel-wise error can be displayed as a topographic map, showing the accuracy of estimations in the spatial domain across multiple channels. In addition, the spatial collective average over a set Λ is denoted by $ave\;nRMSE=\frac{1}{\left|\Lambda \right|}\sum_{i\in \Lambda }nRMSE_{i}$.

### 5.1. Performance evaluation on EEG reconstruction

#### 5.1.1. EEG reconstruction results from synthesized data

**Figure 4:**
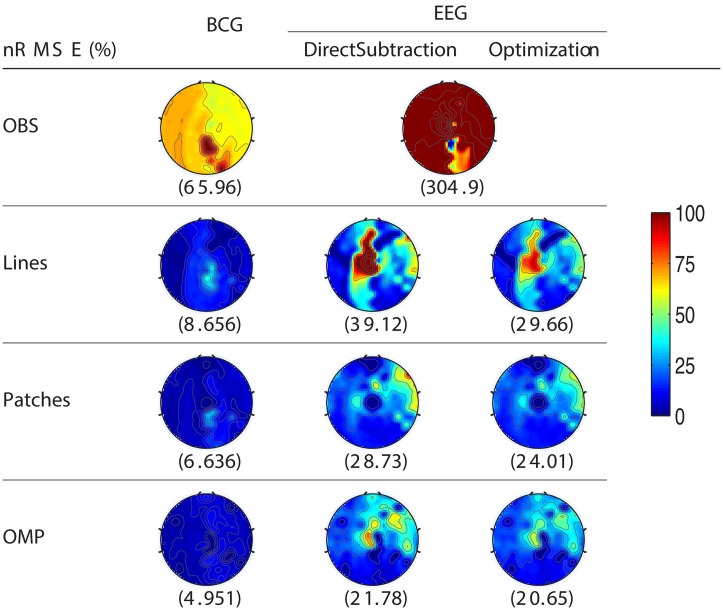
**Topographic maps of *nRMSE*(%) after averaging all cross-validation results**. The spatially collective *ave nRMSE*s(%) over all channels are in the brackets.

**Figure 5:**
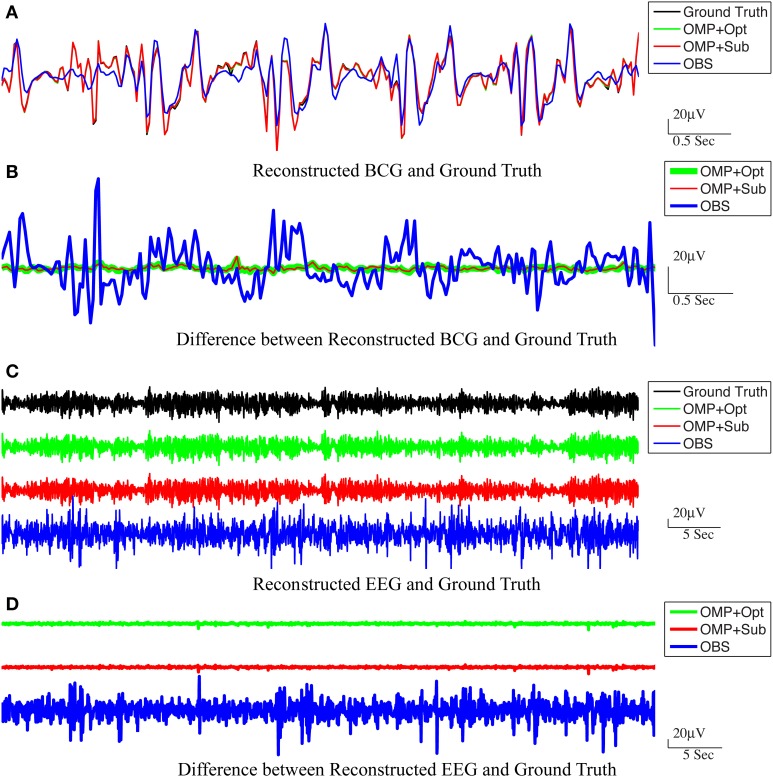
**Reconstructed BCG (A) and EEG (C) from channel 118**. In **(A,C)**, the top panel: ground-truth used in simulation. From second to bottom panels, the methods used to reconstruct the signals are OMP with direct subtraction, OMP with Optimization-based reconstruction and OBS. The difference between the reconstructed signals and the ground truth are displayed in **(B,D)** for BCG and EEG signals, respectively.

**Figure 6:**
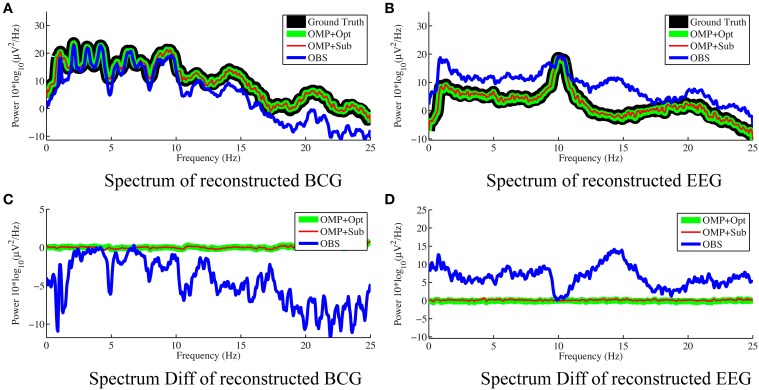
**Frequency spectra of reconstructed BCG (A) and EEG (B) from channel 118 with 0.05 Hz frequency resolution**. The OMP related approaches reconstruct signals that are so close to the ground truth that their spectra almost perfectly overlap. The subtractions of the spectra between reconstructed signals and ground truth are displayed in **(C,D)** for BCG and EEG signals, respectively.

For the purpose of evaluating the overfitting and consistency of our proposed framework, the 12-min long synthesized data were partitioned further into three equal size datasets for cross-validation, in which parameters were selected by a grid-search over parameter space from training datasets and tested on the validation datasets. The topographic maps of *nRMSE* averaged from the validation datasets are reported in Figure 4 with their collective averages *ave nRMSE* in the brackets. Our methods show improvements not only in a few selected channels but across the topology of the scalp. In addition, the temporal and spectral plots of ground-truth and reconstructed BCG and EEG signals appear in Figures 5, 6.

**Figure 7:**
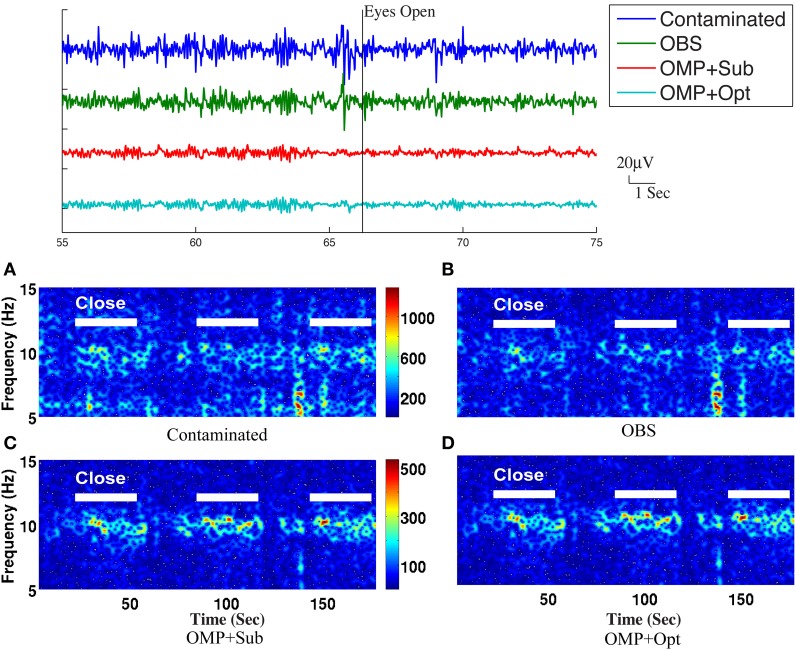
**Top panel:** Roughly 20-s ranges of reconstructed EEG signals are shown here with event onsets (eyes open and eyes close). **Bottom panel:** Comparison of performance in differentiating the eyes open (EC) and eyes closed (EO) states: **(A)** based directly on contaminated EEG recording, **(B)** recovered EEG signals with the OBS method, and **(C)** the OMP with direct subtraction and **(D)** the OMP with optimization-based method. The left panel depicts the reconstructed EEG spectrograms.

As shown in Figure 4, the best result, obtained by combining OMP approach with the optimization-based reconstruction, offers approximately 14.6-fold improvement compared to OBS in full-scalp EEG reconstruction. In comparison, our previous results using neighboring channel as BCG prior reported only 7 fold improvement. Even the simple direct subtraction with two *ad hoc* patterns can improve the EEG reconstruction quality by 10 to 12-fold. We interpret the overall reduced energy in the spectrum and spectrogram of reconstructed data in Figures 6B, 7 as a reflection of the artifact contribution. All spectrograms were produced with 0.1 Hz frequency resolution, a Hamming window of length 256 and the number of points that each segment overlaps being 200.

#### 5.1.2. EEG reconstruction results from real contaminated measurements

Because it is not possible to measure ground-truth BCG and EEG components from uninsulated channels inside the scanner, the performance of EEG reconstruction can only be examined qualitatively for known EEG features. One important feature is the increased power in the alpha band (8–10 Hz), primarily in the posterior electrodes, when the subjects eyes are closed (EC), compared to the eyes open (EO) condition. We collected recordings from 20 unblocked channels with BCG contamination, arranged according to the conventional 10-20 system, when three subjects were cued verbally to open and close their eyes every 30 s for a total time of 14 min. The same protocol was used to collect EEG signals outside the scanner as well, for optimization-based reconstruction, as explained in our previous work (Xia et al., 2013a).

We followed the procedure of Chen et al. (2008) to quantify the EC/EO effects. Each 30-s EEG sample, omitting 3-s before and after each EC/EO event onset, was analyzed in 3 s epochs, resulting in 112 epochs for each EC/EO state. The absolute EEG band power (μ*V*^2^) in the alpha band from each epoch of EC/EO state was calculated using the Fast Fourier Transform. As the alpha band power values failed a normality test, the Wilcoxon test for nonparametric comparison of ranks was performed, with *p*<0.05 accepted as significant, to assess the hypothesis that EC and EO states have similar population mean rank based on alpha band power (Chen et al., 2008).

The top panel of Figure 7 illustrates qualitatively the experimentally acquired contaminated data from an occipital channel (channel 124) from one of the subjects, and the corresponding reconstructed EEG signals from OBS, OMP inference with direct subtraction and optimization-based reconstruction methods. The transition from EC to EO states are clearly identifiable at around 65 s. EEG signals reconstructed with OMP inference model have revealed better-preserved alpha rhythm in EC state than OBS.

The spectrogram of recovered EEG from one subject are shown in the bottom panel of Figure 7 for quantitative comparisons while statistical results from all subjects are presented in Table 1. With 112 epochs, the Wilcoxon-test on the contaminated data indicates no marked reduction in the magnitude of alpha band power in the EO states. Agreeing with the results reported in Niazy et al. (2005), statistically significant difference in alpha band power is present between the EC and EO states of the recovered EEG signals from the OBS. As expected, a more significant statistical difference is revealed using the OMP inference plus direct subtraction, and even greater difference is reported with OMP plus optimization-based reconstruction, in accordance with our results in the simulation study. Similar results are obtained from other subjects and are presented in the Supplementary Material.

### 5.2. Performance evaluation of the inference model

For the purpose of efficiency and stability, it is desirable to learn the inference matrix, **W**, from a short full-scalp BCG-only dataset while maintaining high estimation accuracy. To this end, we first acquired 13-min full-scalp BCG-only recordings to assess the impact of the model building length on the estimation accuracy. We then derived inference matrices from data that varied from 1 min to 4 min in the model building stage, and evaluated the corresponding full-scalp BCG recovery errors (*nRMSE*) on the remainder. We have observed that the duration of model building has negligible effect of less than 0.3%. Hence the results in this paper are reported with the inference matrix from 1 min model building length.

#### 5.2.1. Consistency test results for single and multiple subjects

**Figure 8:**
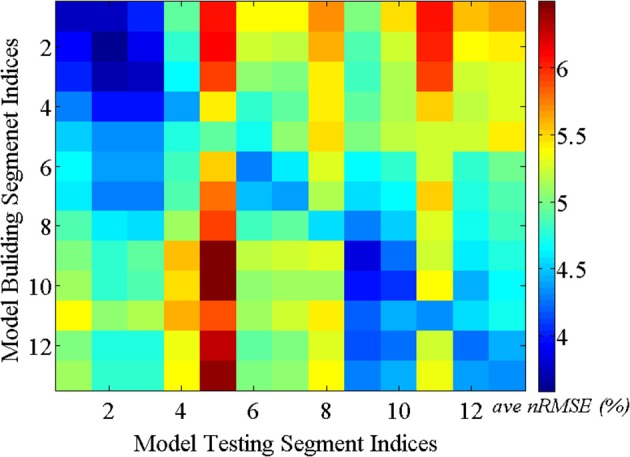
**BCG estimation errors (*ave nRMSE*) in percentage (%) exhibit (sub)diagonal structure for models built on one segment and applied to another segment**.

***5.2.1.1. Consistency test over time***. As discussed above, it is reasonable to expect that the spatial correlations of BCG traces among multiple channels remain consistent over time. This assumption ensures acceptable BCG estimation over time under our inference model. We validated this presumption using the following steps: First, a total of 13-min full-scalp BCG-only recordings were partitioned into 13 equal length segments. Then, the inference matrix built from each segment (training segment) was evaluated on each of the remainder segments (testing segments), forming an error matrix of spatially collective average *ave nRMSE* whose *(i,j)^th^* entry contains the *ave nRMSE* of segment *j* in column direction based on the model built on segment *i* along row direction. The evolution of the inference relationship is visualized in Figure 8. As expected, the (sub)diagonal structure of the error matrix in Figure 8 suggests mild non-stationarity, but with a uniform upper bound of less than 10%, confirming the presence of a generally stable inference relationship.

***5.2.1.2. Channel consistency test***. We performed a similar validation testing the representativeness of selected channels on full-scalp 5-min BCG-only recordings from three experimental sessions of the same subjects, and three sessions of 9-min recordings from three subjects. Error matrices were generated after dividing each recording into 1-min long segments. Mean values of the error matrices are presented in Channel Consistency column of both Tables 2, 3, while the raw error matrices of all consistency tests are shown in the Supplementary Material. Highest accuracy is achieved in general when the training and testing segments are from the same session or subject, emphasizing the necessity of subject-specific channel selection.

**Table 2:** **Mean values of the error matrices whose element is the BCG estimation error (*ave nRMSE*) in percentage (%) for a model whose subset of channels and inference matrix were learned from one training segment and applied to another testing segment**.

**%**	**Channel consistency**			**Training inference**			**Recalculated inference**		
	**Session 1**	**Session 2**	**Session 3**	**Session 1**	**Session 2**	**Session 3**	**Session 1**	**Session 2**	**Session 3**
Session 1	3.98	7.56	5.10	4.02	46.69	38.67	4.59	8.50	5.48
Session 2	4.98	6.52	4.90	33.14	6.85	30.76	5.87	7.33	5.28
Session 3	4.33	7.25	4.44	31.02	36.82	4.53	5.07	8.06	4.79

*Data were from three different experimental sessions. Three columns represent three different conditions for estimating the BCG of the testing segments. Channel Consistency column: an optimal inference matrix was used in estimation. Training Inference column: both channels and inference matrix were from the training data. Recalculated Inference column: Inference matrix was updated with the proposed recalculation method. (All error matrices are presented in the Supplementary Material)*.

**Table 3:** **The same as Table 2 but data were from three subjects**.

**%**	**Channel consistency**			**Training inference**			**Recalculated inference**		
	**Session 1**	**Session 2**	**Session 3**	**Session 1**	**Session 2**	**Session 3**	**Session 1**	**Session 2**	**Session 3**
Session 1	3.92	5.43	5.51	4.11	38.88	38.23	4.44	6.49	6.81
Session 2	5.01	4.68	5.63	41.94	4.83	50.15	5.83	5.44	6.72
Session 3	5.36	5.63	4.78	47.32	48.13	5.04	6.24	6.62	5.51

An optimal inference matrix, built directly from the testing rather than the training segment, was applied in each testing segment estimation using the channels selected from the training segment. With less than 2% increase in errors (from the consistent errors for each testing data), the selected channels are representative not only for different experiment sessions but also for different subjects.

***5.2.1.3. Consistency test with inference matrix from training***. Without applying the optimal inference matrix, we obtained the mean values from the error matrices when the channels and inference matrix were both from the training data and presented the result in Training Inference column of both Tables 2, 3. Combining the results from Channel Consistency column and Training Inference column, it is safe to conclude that the observed excessive errors (in Training Inference column) should originate mainly from the inconsistency of inference matrix rather than from the selecting channels failing to be representative. This observation also helps us to determine a fixed blocking pattern, especially when lower BCG-only estimation accuracy is tolerable; in practice this might greatly reduce experiment time and complexity

***5.2.1.4. Consistency test with recalculated inference matrix***. Furthermore, the application of our inference matrix recalculation method decreases the errors to reasonable levels (most errors are approximately bounded above by 10%) as illustrated in Tables 2, 3 (Recalculated Inference column), agreeing with the theoretical proof in the Supplementary Material.

#### 5.2.2. Determination of “budget” size

**Figure 9:**
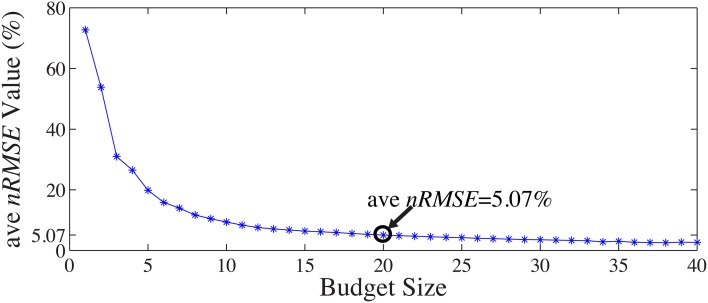
**Mean *ave nRMSE*(%) of 12 validation sets as a function of “budget” size**.

A 13-fold cross-validation was employed to determine the size of the subset Λ_*ins*_ with 13 one-minute segments from the 13-min full-scalp BCG-only signals: for each test in the k-fold process, the 1 min training data from the *k^th^*-minute BCG signals was denoted as **X***^(k)^_bcg_*, and the validation data from the remaining 12-min was denoted as **X***^(k)^_bcg_*. The inference matrix **W** was built first by solving Equation 3. on the training data with a specific “budget” size, and then applied on the validation set to estimate the BCG components from the non-insulated channels. Spatial collective average errors from estimating the validation sets were calculated for different folds, and for different “budget” sizes. The averaged *ave nRMSE*s over 13 different validation sets decreases as the “budget” size increases, as shown in Figure 9. In this paper, we chose |Λ_*ins*_| to be 20 as we determined that a consistent 5% estimation error is acceptable.

#### 5.2.3. Inference performance of “random” patterns

**Figure 10:**
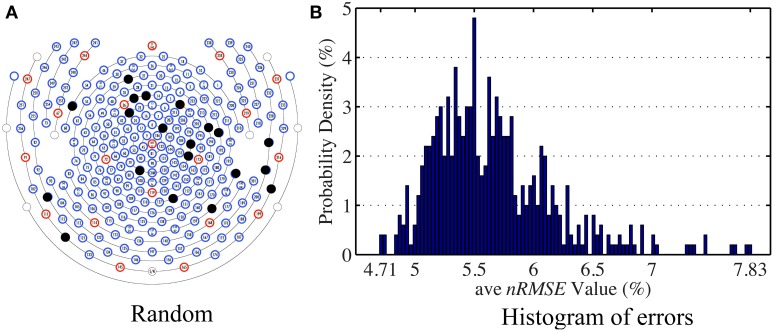
**(A)** One of the patterns selected with random permutation. **(B)** Histogram of the estimation errors (*ave nRMSE*) from 500 realizations of random patterns. The highest error is 7.83% when the lowest error is 4.71%.

**Figure 11:**
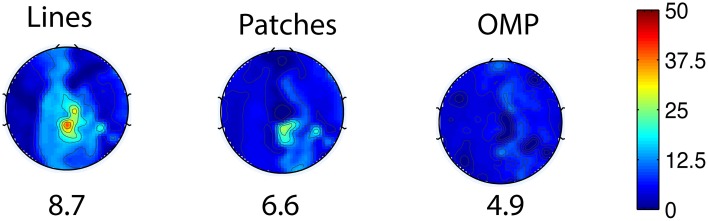
**Topographic BCG estimation error (*nRMSE*) maps in percentage (%) for different channel selection methods when the model building duration is 1 min**. The number at the bottom of each error map: the *ave nRMSE* averaged spatially across all channels.

The general set selection problem resembles “set selection” problems in compressed sensing, which maximizes the ℓ_2_ fidelity (inference goal here) subject to ℓ_0_ constraint (the cardinality of Λ_*ins*_). In practice, the Restricted Isometry Property-type (Candes and Tao, 2005) conditions are hard to verify, especially with the high variation in BCG. However, it would be desirable to obtain insights from reconstruction performance based on randomly selected subsets. To this end, we repeated the channel selection process, drawing |Λ_ins_| = 20 channels from Λ_*full*_ with random permutation. Figure 10A illustrates one of the “random” patterns and Figure 10B shows a histogram of the errors (*ave nRMSE*) corresponding to 500 “random” patterns. The average of *ave nRMSE* over 500 realizations is 5.65% with a maximum error at 7.85% and a minimum at 4.7%. Figure 11 reports the topographic maps of *nRMSE* when the model building length is 1 min, and shows that the OMP approach achieves better BCG estimation performance than the other two *ad hoc* insulation patterns. Comparing Figures 10, 11, we notice that the “random” pattern consistently performs better than the “lines” pattern. On average, the “random” pattern performs better than the “patches” pattern and worse than the pattern from the OMP approach. As can be observed, only a few instances would result in estimation accuracies higher than the one from the OMP approach who yields consistently good estimation results.

## 6. Discussions and conclusion

With increased magnitude at higher MR field strengths and high temporal non-stationarity, BCG artifacts have impeded further investigations using concurrent EEG-fMRI. Previous solutions to this problem focus mainly on PCA or ICA-based approaches, addressing this issue by channel-wise temporal modeling of BCG. To take full advantage of the spatial information from a high-density EEG cap, it is natural to extend to spatial PCA/ICA (Bénar et al., 2003; Srivastava et al., 2005). However, the success of modeling BCG with such spatial PCA/ICA approaches demands the existence of questionable (Grouiller et al., 2007; Vanderperren et al., 2010) orthogonality or independence relationship between the full-scalp BCG and EEG signals. By contrast, our approach imposes no presumptions on the relationship between BCG and EEG, and relies on a consistent inference model that maps BCG from a subset of channels to BCG from all channels. With 20 degrees of freedom, the inference model produces an observation-space-to-observation-space map that is robust to variations in BCG source space. Unlike spatial PCA/ICA that requires prior selection of the number of components or subjective identification of components, our estimation of BCG is based on the widely accepted additive generative model.

Extending from our previous work, which used the BCG recordings from subjectively-selected insulated neighboring channels, the present study: (1) estimates the full-scalp BCG components for all channels from an automatically selected insulation set; (2) reconstructs all channels at once with significantly improve quality since it relies on stable global rather than ad-hoc local neighboring BCG information; (3) simplifies the experimental setup by insulating only a small subset of channels. The inference model has improved the estimation accuracy of BCG greatly across all channels, since the inference relationship is generally consistent in time and the selected channels are reasonably consistent not only across experimental sessions but also across subjects, suggesting that the subject-specific channel selection is less essential when some inaccuracy of BCG estimation accuracy is tolerated. In spite of the observed rather significant inconsistency of the inference matrix across sessions, our proposed inference matrix recalculation method effectively keeps the errors below 10% upper bound which is only approximately 1–2% worse in comparison to those where optimal inference matrices were adopted.

In practice, our inference model involving only linear regressions is easy to build and calculate, with a few minutes of experimental time for model-building stage I, and with no hardware modifications, unlike other methods (Dyrholm et al., 2009) utilizing multiple channels of EEG cap. In addition, while we developed our method on a high density cap, it can be applied to a lower density cap with some variations in BCG reconstruction accuracy. Once the inference matrix is learned, the mitigation of BCG artifacts, with our inference approach and direct subtraction-based reconstruction, is suitable for on-line artifact removal, requiring only multiplication and subtraction that can be performed in real-time. Note that the direct subtraction approach in Equation 6 works best when the observation noise ε in Equation 1 is relatively low. In realistic measurement settings, the noise level is not accessible directly, and can be estimated only under certain distributional assumptions. Practically, this seems to be reasonable: we have observed that subtraction-based reconstruction works well on both synthesized and real contaminated data. For localized inference of interest, e.g., occipital channels for alpha rhythm studies, the proposed method can be modified trivially (the regression goal in Equation 3) to for selective optimization.

Moreover, our method can be integrated with these approaches, such as the KappaMetrics fEEG^TM^ system and others (Bonmassar et al., 2002; Masterton et al., 2007; Chowdhury et al., 2014), that generate BCG reference signals, providing guidance for placements of motion sensors, wire loops and fewer number of channels for the reference layer. Those reference-based methods are attractive however require specialized hardware. For example we were not able to compare it directly because it is not available. Admittedly, there exists some discrepancy between each of these reference signals and the “ground-truth” BCG signals, as a result of insulation, sensing process or impedance mismatch. As suggested by others (Ullsperger and Debener, 2010), these signal differences may become the limiting error term when used simply for linear subtraction thus necessitating further correction methods. Our method compensates for such signal differences in two ways. First, the proposed inference matrix recalculation method learns the updated BCG spatial relationship from contaminated data, effectively minimizing the discrepancy between the reference signal and BCG component of contaminated data. Second, our method adopts an optimization-based EEG reconstruction scheme to further reduce residual BCG signals after subtraction. In principle, Hall effects (Yan et al., 2010; Mullinger et al., 2013; Chowdhury et al., 2014) occurring in the MR imaging field might distort the scalp topography of the EEG signals. It is difficult to estimate the magnitude of this contaminant, which is common to OBS and other reference signal based methods.

This paper has focused mainly on removing BCG signals for non-ERP studies. Although our method in principle extends to ERP studies, the artifact suppression effects may not significantly outperform the OBS method, as shown in Supplementary Material, due to the fact that averaging around known triggering events will reduce BCG residual signals when event timing is not correlated to the heartbeats. A crucial result reported here, is that our method is robust in exposing alpha power fluctuations under experimental conditions. In our hands this had been a difficult challenge when using other artifact removal methods, and has limited sharply the value of combined EEG-fMRI experiments that seek to look at continuously recorded signals and to analyze their spectral content.

Combined, the proposed framework can do much to mitigate the serious artifacts that otherwise limit combined EEG-fMRI recordings. The practical advantage of doing so may be very large. While many groups have shown important results of combined EEG-fMRI in the event-related designs that are relatively resistant to the BCG artifacts; few reports show success in continuous recordings. The latter, however, are necessary to study the tantalizing relationships between BOLD signal and brain EEG rhythms, as well as important disease entities such as epilepsy, where there is little opportunity to average EEG events.

### Conflict of interest statement

The authors declare that the research was conducted in the absence of any commercial or financial relationships that could be construed as a potential conflict of interest.
