# Supplementary material for: Removing ballistocardiogram (BCG) artifact from full-scalp EEG acquired inside the MR scanner with Orthogonal Matching Pursuit (OMP)
Source: Front Neurosci. 2014 Jul 29;8:218. doi: 10.3389/fnins.2014.00218 (PMC4114198; doi:10.3389/fnins.2014.00218)
Supplement: Supplementary file 1 [file Presentation1.PDF]

Frontiers in Brain Imaging Methods

Original Research Article
29 April 2014

Supplementary Material: Removing ballistocardiogram (BCG) artifact from full-scalp EEG acquired inside the MR scanner with Orthogonal Matching Pursuit (OMP)

Hongjing Xia [1,*], **Dan Ruan** [1,2] **and Mark S. Cohen** [1,3]

[1] *Department of Bioengineering, University of California, Los Angeles, CA, USA*
[2] *Department of Radiation Oncology, University of California, Los Angeles, CA, USA*
[3] *Department of Psychiatry, Neurology, Radiology Biomedical Physics and Psychology, University of California, Los Angeles, CA, USA*

Correspondence*:
Hongjing Xia
Department of Bioengineering, B8-169 Semel Neuropsychiatric Institute, University of California, Los Angles, 760 Westwood Plaza, Los Angeles, CA 90095-8353, USA, xiahongjing@ucla.edu

1 PROOF OF THE INFERENCE MATRIX RECALCULATION METHOD

We denote the contaminated data as $\mathbf{Y} \in \mathbb{R}^{C \times T}$, with C being the number of channels and T being the number of time points of the recordings. The time points are further decomposed into K segments with L points each according to some integer number of heartbeat cycles.

Averaging of all the segments along the time direction is equivalent to right multiplying a circulant matrix $\mathbf{A} \in \mathbb{R}^{KL \times L}$ formed from a scalar $\frac{1}{K}$ and vertically stacking K identity matrices $\mathbb{1}$ with length L:

$$\mathbb{1} = \begin{pmatrix} 1 & 0 & \cdots & 0 \\ 0 & 1 & \cdots & 0 \\ \vdots & \vdots & \ddots & \vdots \\ 0 & 0 & \cdots & 1 \end{pmatrix} \in \mathbb{R}^{L \times L}, \mathbf{A} = \frac{1}{K} \begin{pmatrix} 1 & 0 & \cdots & 0 \\ 0 & 1 & \cdots & 0 \\ \vdots & \vdots & \ddots & \vdots \\ 0 & 0 & \cdots & 1 \\ \vdots & \vdots & \vdots & \vdots \\ 1 & 0 & \cdots & 0 \\ 0 & 1 & \cdots & 0 \\ \vdots & \vdots & \ddots & \vdots \\ 0 & 0 & \cdots & 1 \end{pmatrix} = \frac{1}{K} \begin{pmatrix} \mathbb{1} \\ \vdots \\ \mathbb{1} \end{pmatrix} \in \mathbb{R}^{KL \times L}.$$

To maintain the size along the time direction after averaging, the resultant matrix is replicated K times, which is equivalent to right multiplying another circulant matrix formed from stacking identify matrices with length L horizontally. Combined, averaging and replicating the segments is equivalent to right

multiplying a circulant matrix $\mathbf{C} \in \mathbb{R}^{KL \times KL}$:

$$\mathbf{C} = \frac{1}{K} \begin{pmatrix} \mathbb{1} & \cdots & \mathbb{1} \\ \vdots & \ddots & \vdots \\ \mathbb{1} & \cdots & \mathbb{1} \end{pmatrix} \in \mathbb{R}^{KL \times KL}.$$

Naturally, we have the following relations:

$$\begin{aligned} \bar{\mathbf{Y}} &= \mathbf{YC}, \\ \bar{\mathbf{X}}_{bcg} &= \mathbf{X}_{bcg}\mathbf{C}, \\ \bar{\mathbf{X}}_{eeg} &= \mathbf{X}_{eeg}\mathbf{C}, \\ \bar{\mathbf{Y}} &= \bar{\mathbf{X}}_{bcg} + \bar{\mathbf{X}}_{eeg} \in \mathbb{R}^{C \times KL}, \end{aligned} \tag{1}$$

As in previous papers by **Niazy et al.** (2005); **Allen et al.** (2000), we assume that EEG becomes uncorrelated after 3 sec. Choosing K and L properly, we assume $\bar{\mathbf{X}}_{eeg} \to 0$ and $\bar{\mathbf{Y}}$ becomes a good representation of $\bar{\mathbf{X}}_{bcg}$ as as result of the averaging.

Ideally, we derive our inference matrix from the following:

$$\widetilde{\mathbf{W}} = \arg\min_{\widetilde{\mathbf{W}}} \left\| \mathbf{X}_{bcg}[\Lambda_{full}, :] - \widetilde{\mathbf{W}}\mathbf{X}_{bcg}[\Lambda_{ins}, :] \right\|_F^2. \tag{2}$$

We propose to recalculate the inference matrix from the following:

$$\bar{\mathbf{W}} = \arg\min_{\bar{\mathbf{W}}} \left\| \bar{\mathbf{Y}}[\Lambda_{full}, :] - \bar{\mathbf{W}}\mathbf{X}_{bcg}[\Lambda_{ins}, :] \right\|_F^2. \tag{3}$$

We will show that $\bar{\mathbf{W}}$ can recover $\bar{\mathbf{X}}_{bcg}[\Lambda_{full}, :]$ from $\mathbf{X}_{bcg}[\Lambda_{ins}, :]$ with high accuracy. For convenience, we denote $\mathbf{X}_{bcg}[\Lambda_{ins}, :]$ as $\mathbf{X}_0$, $\mathbf{X}_{bcg}[\Lambda_{full}, :]$ as $\mathbf{X}$, and $\bar{\mathbf{X}}_{bcg}[\Lambda_{full}, :]$ as $\bar{\mathbf{X}}$.

After replacing $\bar{\mathbf{Y}}$ with $\bar{\mathbf{X}}_{bcg}$, the solutions to the least square problems above are:

$$\begin{aligned} \widetilde{\mathbf{W}} &= \mathbf{X}\mathbf{X}_0^T(\mathbf{X}_0\mathbf{X}_0^T)^{-1}, \\ \bar{\mathbf{W}} &= \bar{\mathbf{X}}\mathbf{X}_0^T(\mathbf{X}_0\mathbf{X}_0^T)^{-1} = \mathbf{XC}\mathbf{X}_0^T(\mathbf{X}_0\mathbf{X}_0^T)^{-1}, \\ \widetilde{\mathbf{W}} - \bar{\mathbf{W}} &= (\mathbf{X} - \mathbf{XC})\,\mathbf{X}_0^T(\mathbf{X}_0\mathbf{X}_0^T)^{-1}. \end{aligned} \tag{4}$$

The solutions above in Equation 4 minimize the residuals of the least square problems, where these residuals ϵ_1 and ϵ_2 are small quantities:

$$\begin{aligned} \epsilon_1 &= \frac{\left\| \widetilde{\mathbf{W}}\mathbf{X}_0 - \mathbf{X} \right\|_F^2}{\|\mathbf{X}\|_F^2}, \\ \epsilon_2 &= \frac{\left\| \bar{\mathbf{W}}\mathbf{X}_0 - \mathbf{XC} \right\|_F^2}{\|\mathbf{XC}\|_F^2}. \end{aligned} \tag{5}$$

As any circulant matrix can be diagonalized by the Discrete Fourier Transform (DFT) matrix $\mathbf{F}$, we have $\mathbf{C} = \mathbf{F}\boldsymbol{\Lambda}\mathbf{F}^T$. In addition, the eigenvalues of $\mathbf{C}$ are known, and have a certain structure: $\boldsymbol{\Lambda}$ contains

zeros except L ones at its diagonal.

$$\lambda_{i,j} = \begin{cases} 1 & \text{if } i = j \text{ and } i,j \leq L \\ 0 & \text{otherwise} \end{cases}$$

Therefore,

$$\begin{aligned} &\mathbf{XC} = \mathbf{XF\Lambda F}^T \\ &\mathbf{I} - \mathbf{\Lambda} = (\mathbf{I} - \mathbf{\Lambda})^2 = \mathbf{I} - \mathbf{\Lambda}^2 \\ &\|\mathbf{X}\|_F^2 = \mathbf{XX}^T = \mathbf{XFIF}^T = \mathbf{XF}(\mathbf{I} - \mathbf{\Lambda}^2 + \mathbf{\Lambda}^2)\mathbf{F}^T = \mathbf{XF}(\mathbf{I} - \mathbf{\Lambda}^2 + \mathbf{\Lambda}^2)\mathbf{F}^T \\ &= \mathbf{XF}(\mathbf{I} - \mathbf{\Lambda})^2\mathbf{F}^T + \mathbf{XF}(\mathbf{\Lambda})^2\mathbf{F}^T = \|\mathbf{X} - \mathbf{XC}\|_F^2 + \|\mathbf{XC}\|_F^2. \end{aligned} \tag{6}$$

We notice that $\mathbf{XF\Lambda F}^T$ has a physical interpretation. $\mathbf{XF}$ implies application of a discrete fourier transform to each time series (row vector) in $\mathbf{X}$. $\mathbf{XF\Lambda}$ means applying a rectangular low pass filter of length L with a cutoff frequency at $\frac{1}{K} f_s$ where f_s is the sampling frequency. The low-passed version then is transformed to the time domain through the inverse fourier transform by multiplying by $\mathbf{F}^T$. Accordingly, $\|\mathbf{XC}\|_F^2$ represents the energy of the BCG in the low frequency range whose cutoff depends on $\mathbf{\Lambda}$, and the energy in the high frequency range is $\|\mathbf{X} - \mathbf{XC}\|_F^2$. After observing the ground-truth BCG spectrum, as shown in the main body Figure 8, it is reasonable to argue that the percentage of the high frequency energy over the energy of all frequencies $r = \dfrac{\|\mathbf{X} - \mathbf{XC}\|_F^2}{\|\mathbf{X}\|_F^2}$ is a small quantity

The normalized percentage difference of reconstructed signals between the ideal inference matrix and the recalculated one is:

$$\begin{aligned} \frac{\left\|\bar{\mathbf{W}}\mathbf{X}_0 - \widetilde{\mathbf{W}}\mathbf{X}_0\right\|_F^2}{\|\mathbf{X}\|_F^2} &= \frac{\left\|\bar{\mathbf{W}}\mathbf{X}_0 - \mathbf{X} + \mathbf{X} - \widetilde{\mathbf{W}}\mathbf{X}_0\right\|_F^2}{\|\mathbf{X}\|_F^2} \\ &\leq \frac{\|\bar{\mathbf{W}}\mathbf{X}_0 - \mathbf{X}\|_F^2}{\|\mathbf{X}\|_F^2} + \frac{\left\|\mathbf{X} - \widetilde{\mathbf{W}}\mathbf{X}_0\right\|_F^2}{\|\mathbf{X}\|_F^2} \\ &= \frac{\|\bar{\mathbf{W}}\mathbf{X}_0 - \mathbf{X}\|_F^2}{\|\mathbf{X}\|_F^2} + \frac{\left\|\mathbf{X} - \mathbf{XC} + \mathbf{XC} - \widetilde{\mathbf{W}}\mathbf{X}_0\right\|_F^2}{\|\mathbf{X}\|_F^2} \\ &\leq \frac{\|\bar{\mathbf{W}}\mathbf{X}_0 - \mathbf{X}\|_F^2}{\|\mathbf{X}\|_F^2} + \frac{\|\mathbf{X} - \mathbf{XC}\|_F^2}{\|\mathbf{X}\|_F^2} + \frac{\left\|\mathbf{XC} - \widetilde{\mathbf{W}}\mathbf{X}_0\right\|_F^2}{\|\mathbf{X}\|_F^2} \\ &\leq \frac{\|\bar{\mathbf{W}}\mathbf{X}_0 - \mathbf{X}\|_F^2}{\|\mathbf{X}\|_F^2} + \frac{\|\mathbf{X} - \mathbf{XC}\|_F^2}{\|\mathbf{X}\|_F^2} + \frac{\left\|\mathbf{XC} - \widetilde{\mathbf{W}}\mathbf{X}_0\right\|_F^2}{\|\mathbf{XC}\|_F^2} \\ &= \epsilon_1 + r + \epsilon_2. \end{aligned} \tag{7}$$

In sum, since the percentage difference is bounded by the sum of three small quantities, the recalculated inference matrix is indeed a satisfying replacement.

2 SUPPLEMENTARY FIGURES

2.1 FIGURES FOR EXPERIMENTAL SETUP

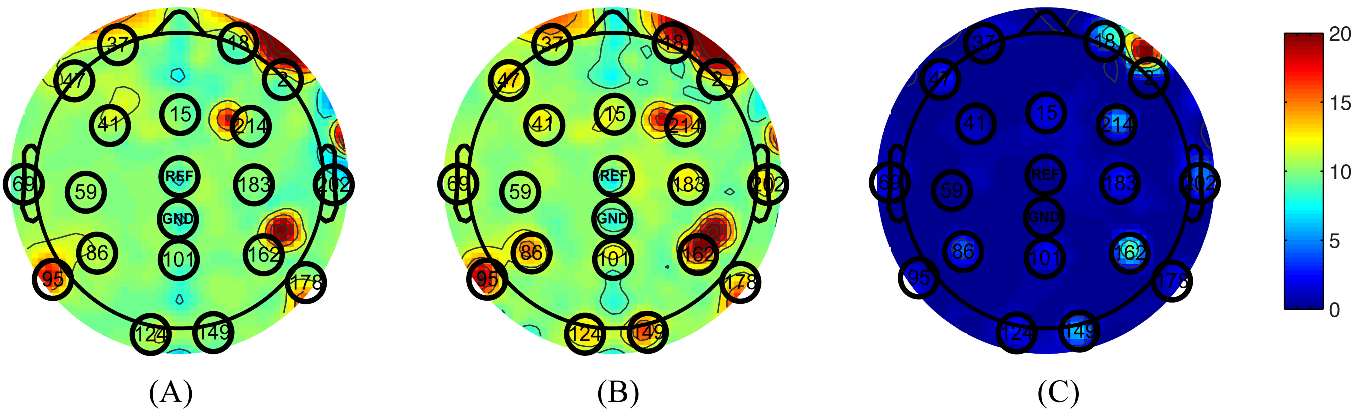

Supplementary Figure 1. The numbers shown on the topographic maps are channel numbers. The conventional channels along with the reference and ground channels are highlighted with black circles. The color indicates the measured impedance number in kΩ. (A) Measured Impedance when all channels are blocked. (B) Measured impedance when reference and ground channels along with conventional 20 channels are unblocked. (C) The difference of impedance between (A) and (B). The average of the difference is approximately 100Ω

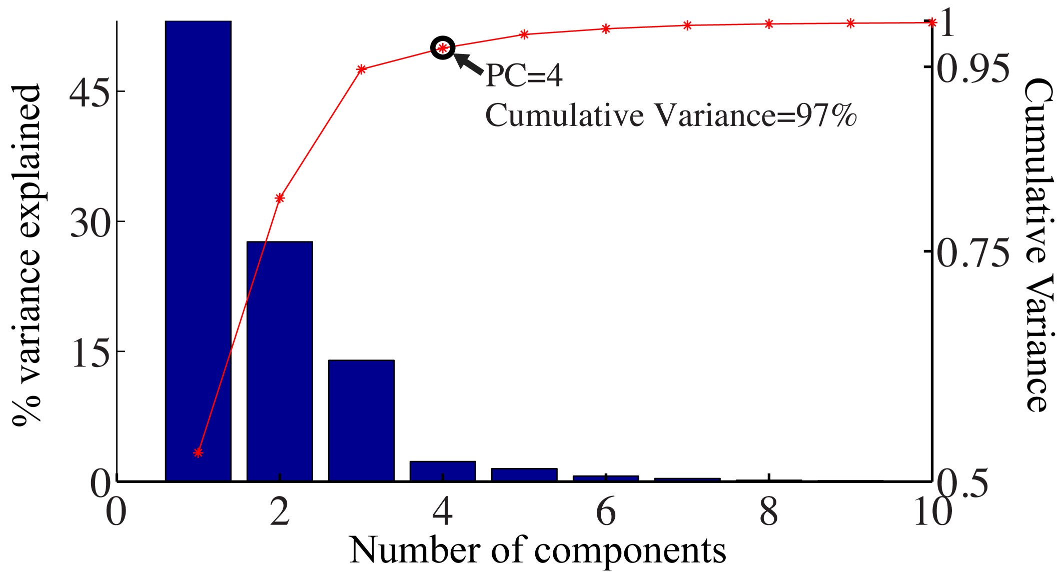

Supplementary Figure 2. The amount of full-scalp BCG variance explained by principal components. Bar plot with ordinate labeling on the left side: the percentage of variance explained by each principal component. Red curve with the ordinate labeling on the right side: The cumulative proportion of the variance explained. The circled value indicates the number of principal components necessary to explain 97% of the total variance.

2.2 FIGURES FOR RESULTS FROM REAL CONTAMINATED EYES OPEN/CLOSE DATA

No significant change in alpha power was detected in the contaminated signal, while the OMP-based and OBS methods display the expected decreases from EC to EO conditions. The left panel of the following figures depicts the reconstructed EEG spectrograms. The right panel displays the Wilcoxon rank test results of alpha band power comparisons between the EO and EC states; standard errors are indicated.

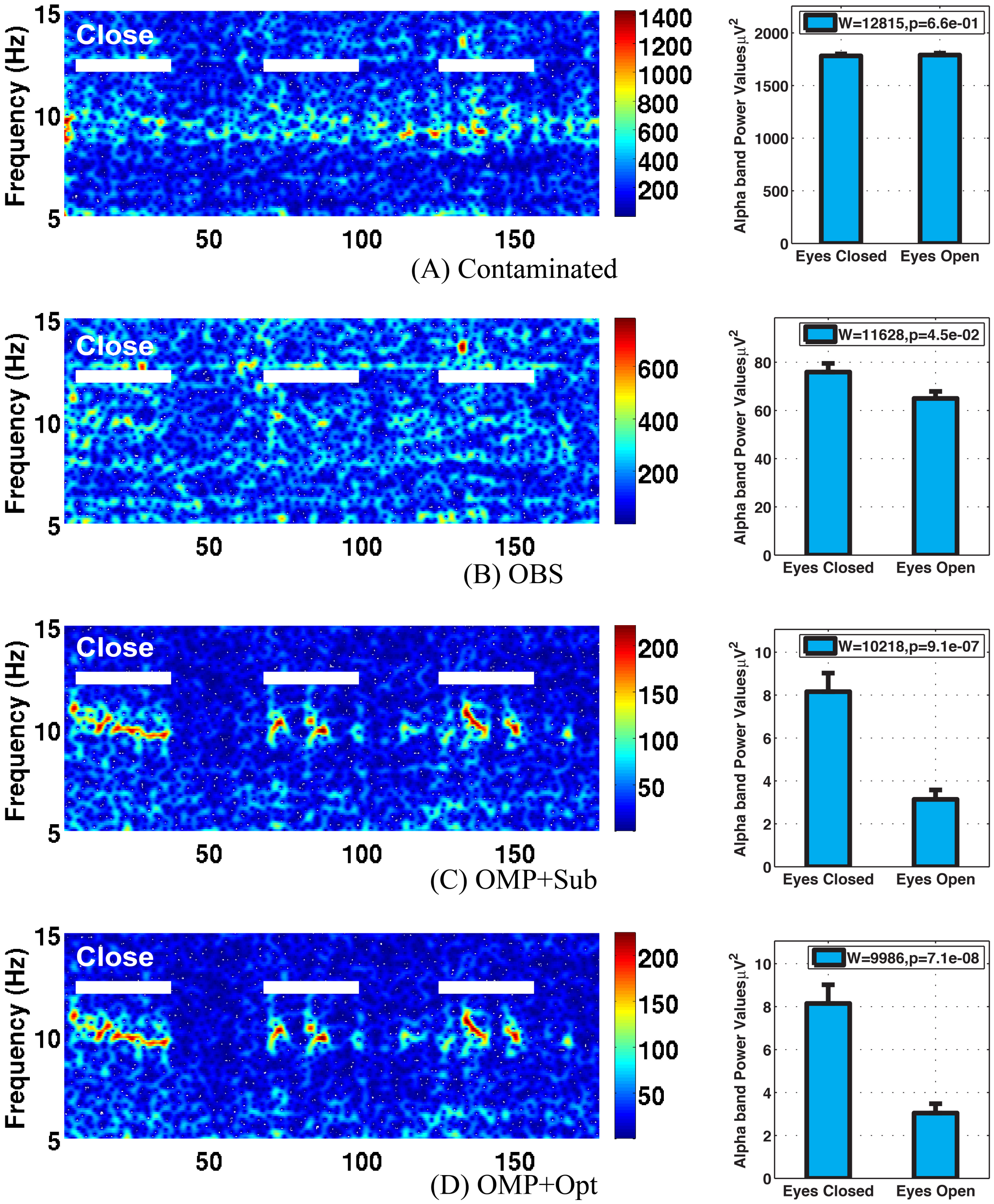

Supplementary Figure 3. Comparison of performance in differentiating the eyes open (EC) and eyes closed (EO) states from **Subject 1**: (A) based directly on contaminated EEG recording, (B) recovered EEG signals with the OBS method, and (C) the OMP with direct subtraction and (D) the OMP with optimization-based method.

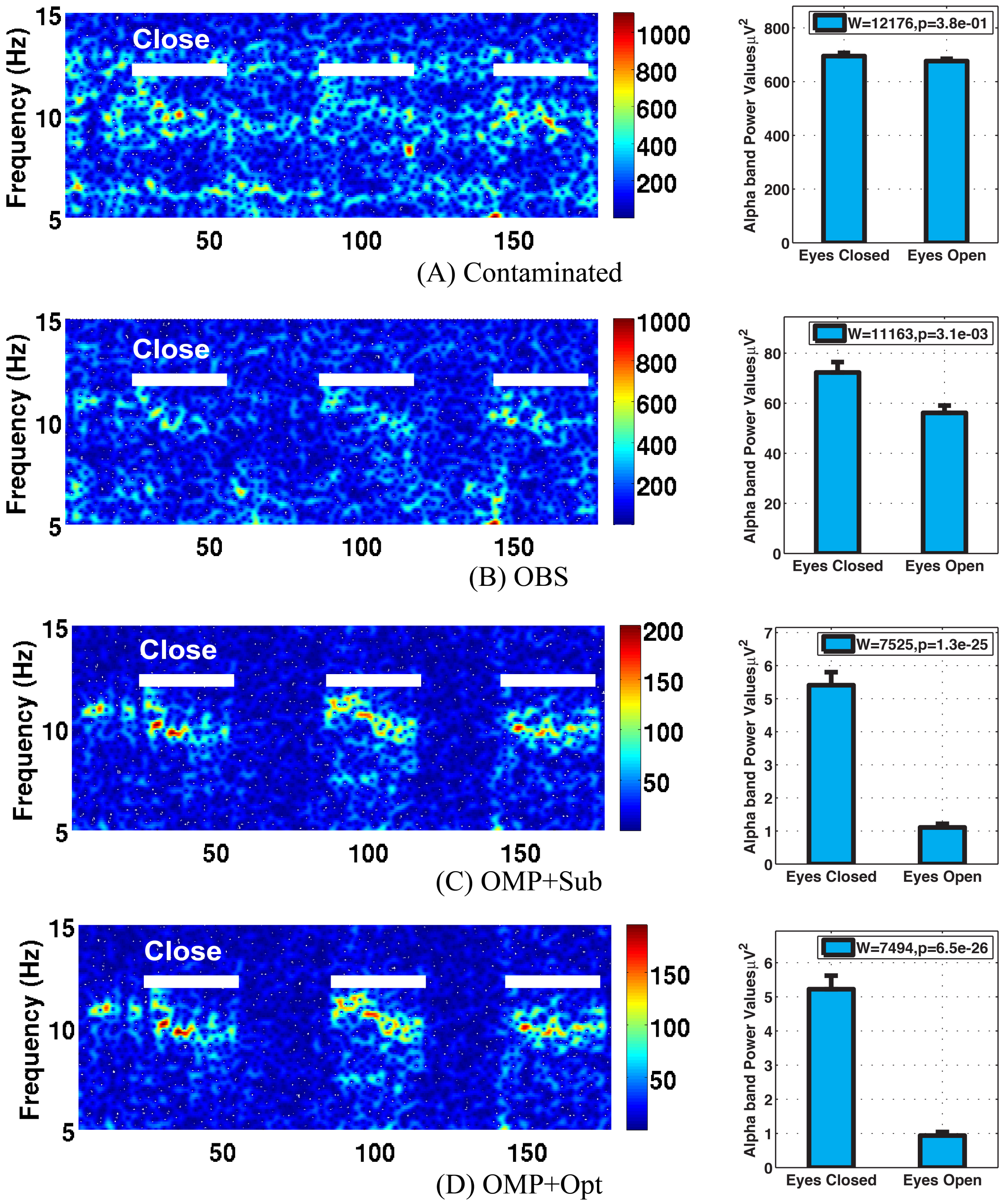

Supplementary Figure 4. The same as Supplementary Figure 3 but from Subject 2

2.3 ERP RESULTS FROM TWO SUBJECTS

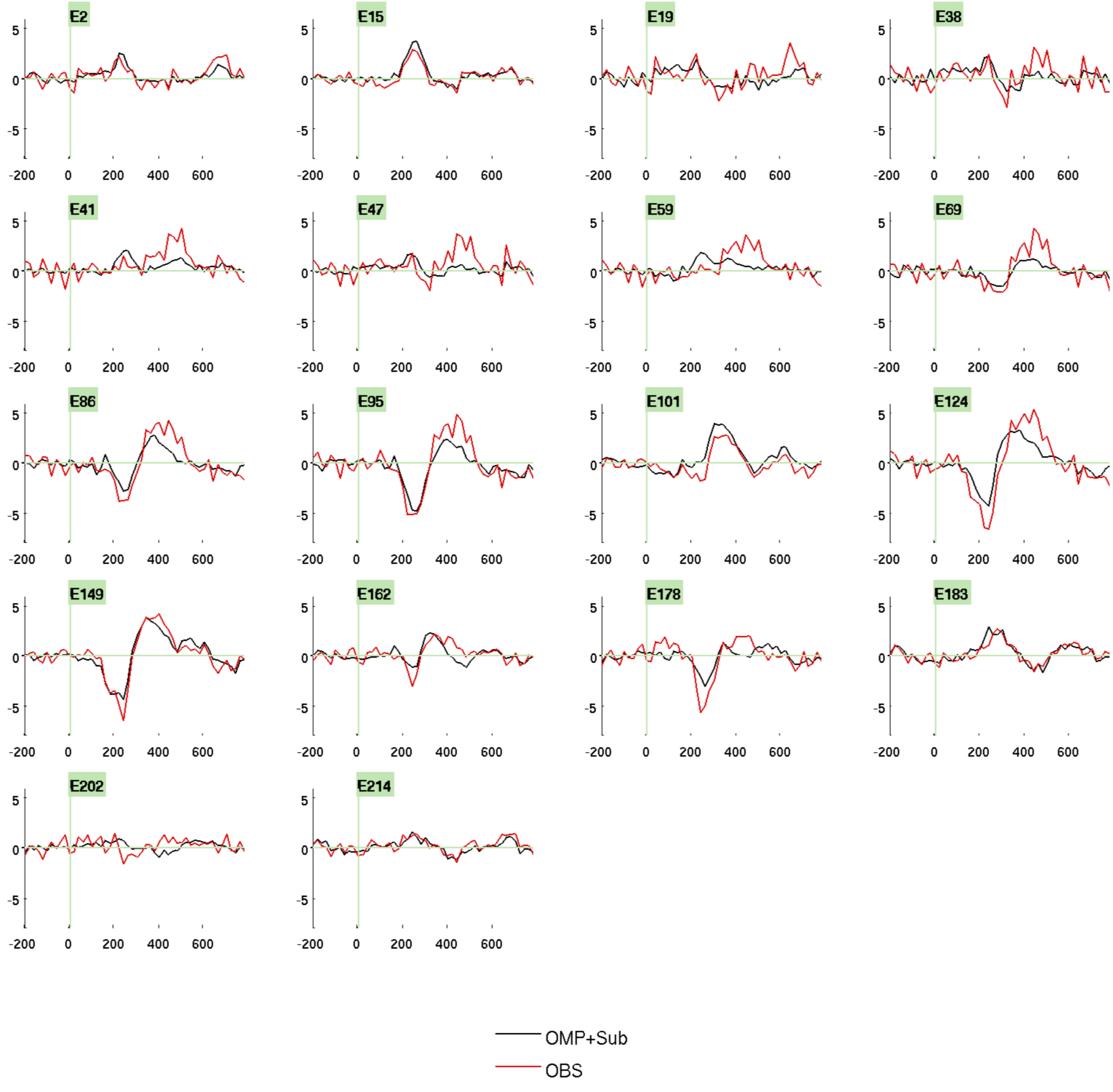

Supplementary Figure 5. Comparison of performance in visual event-related potentials averaged from 150 epochs from **Subject 1**: Black lines represent reconstructed ERPs using the OMP inference method with Direct Subtraction; Red lines represent ERPs when the OBS method is used.

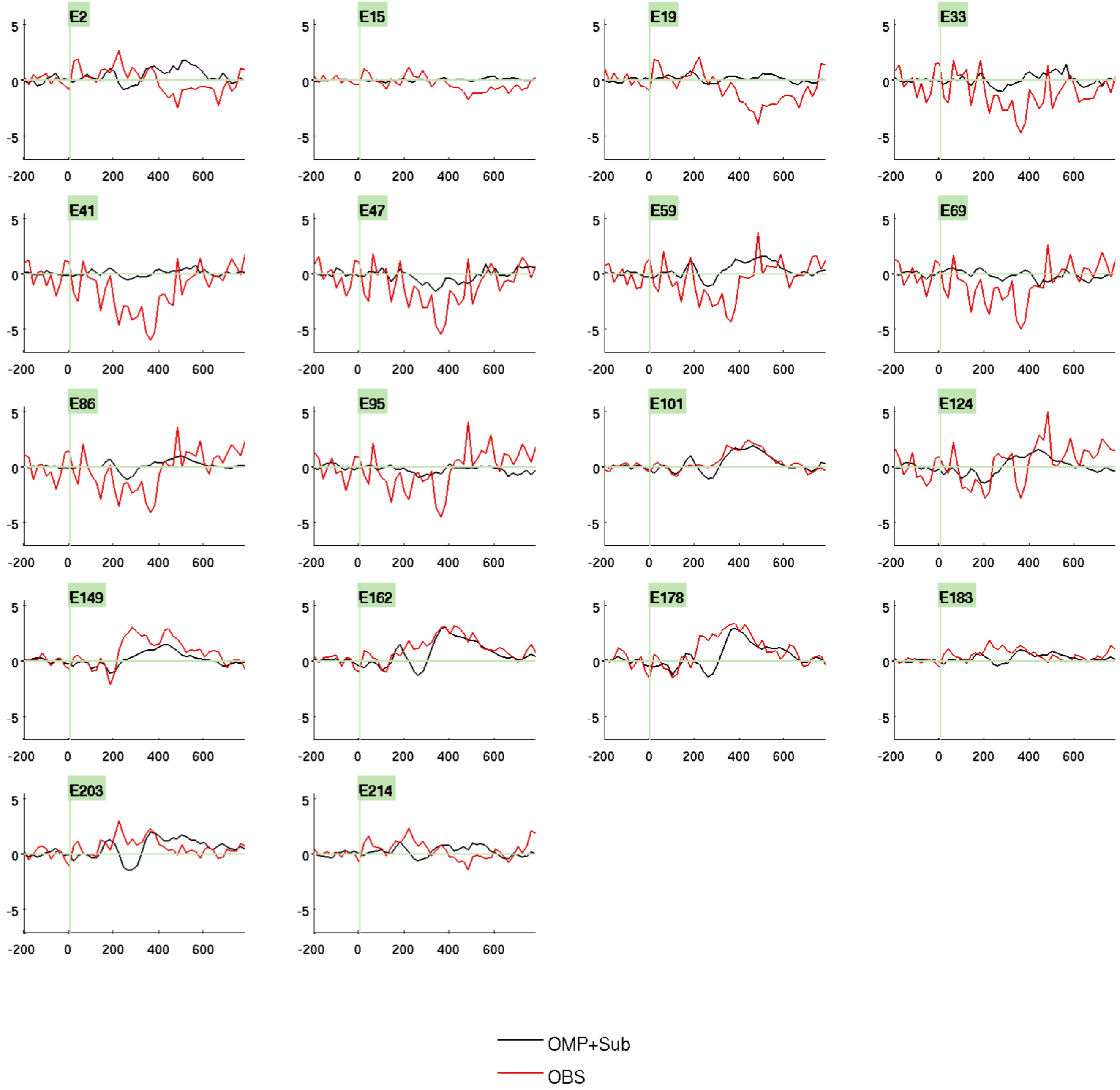

Supplementary Figure 6. The same as Supplementary Figure 5 but from **Subject 2**:

2.4 FIGURES FOR CONSISTENCY TESTS

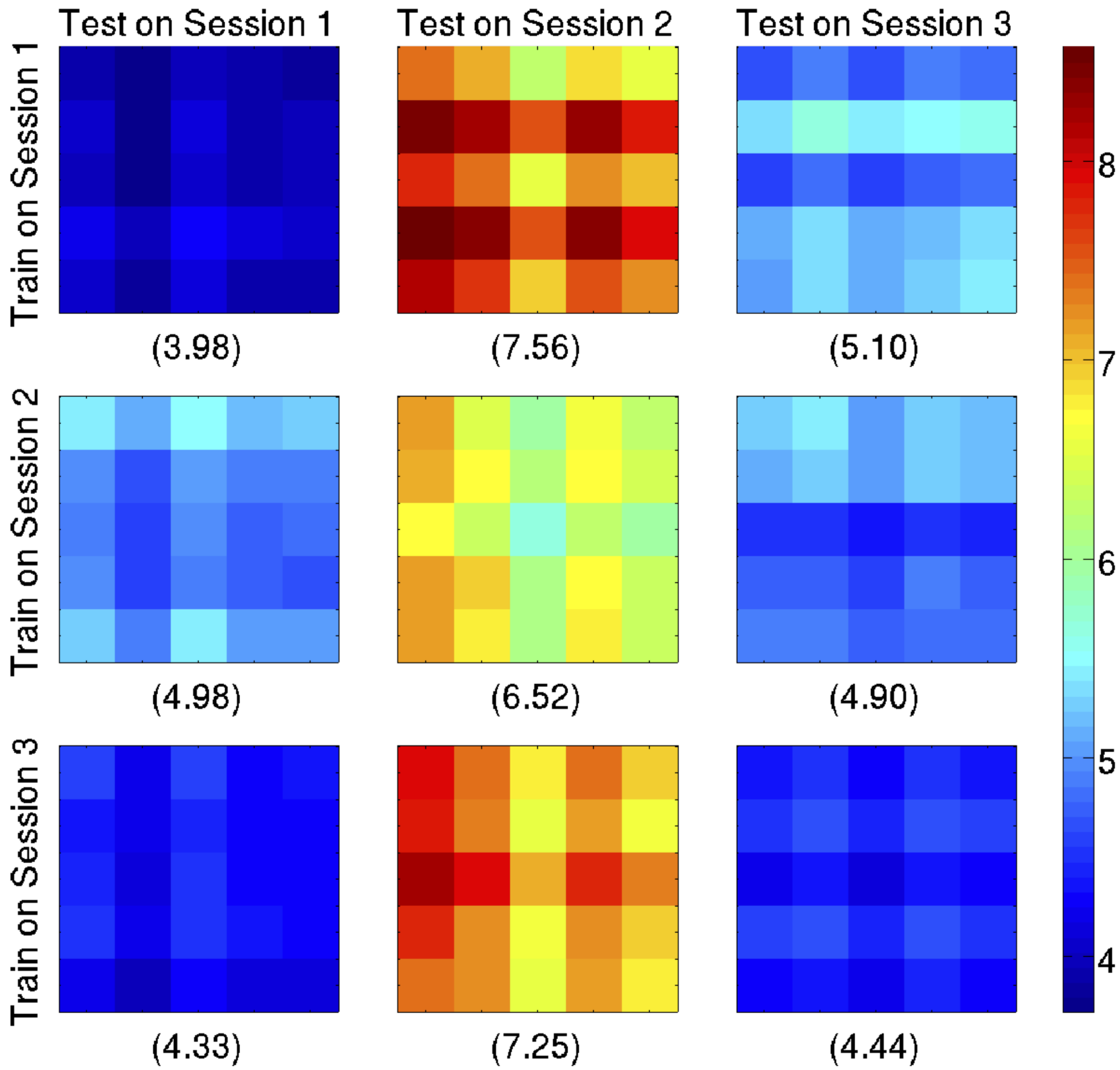

Supplementary Figure 7. BCG estimation errors ($ave\ nRMSE$) in percentage ($\%$) for models whose subset of channels were learned from one training segment and applied to another testing segment of the same subject when the corresponding inference matrix was updated from the testing segment. Mean of each error matrix is shown in the bracket underneath.

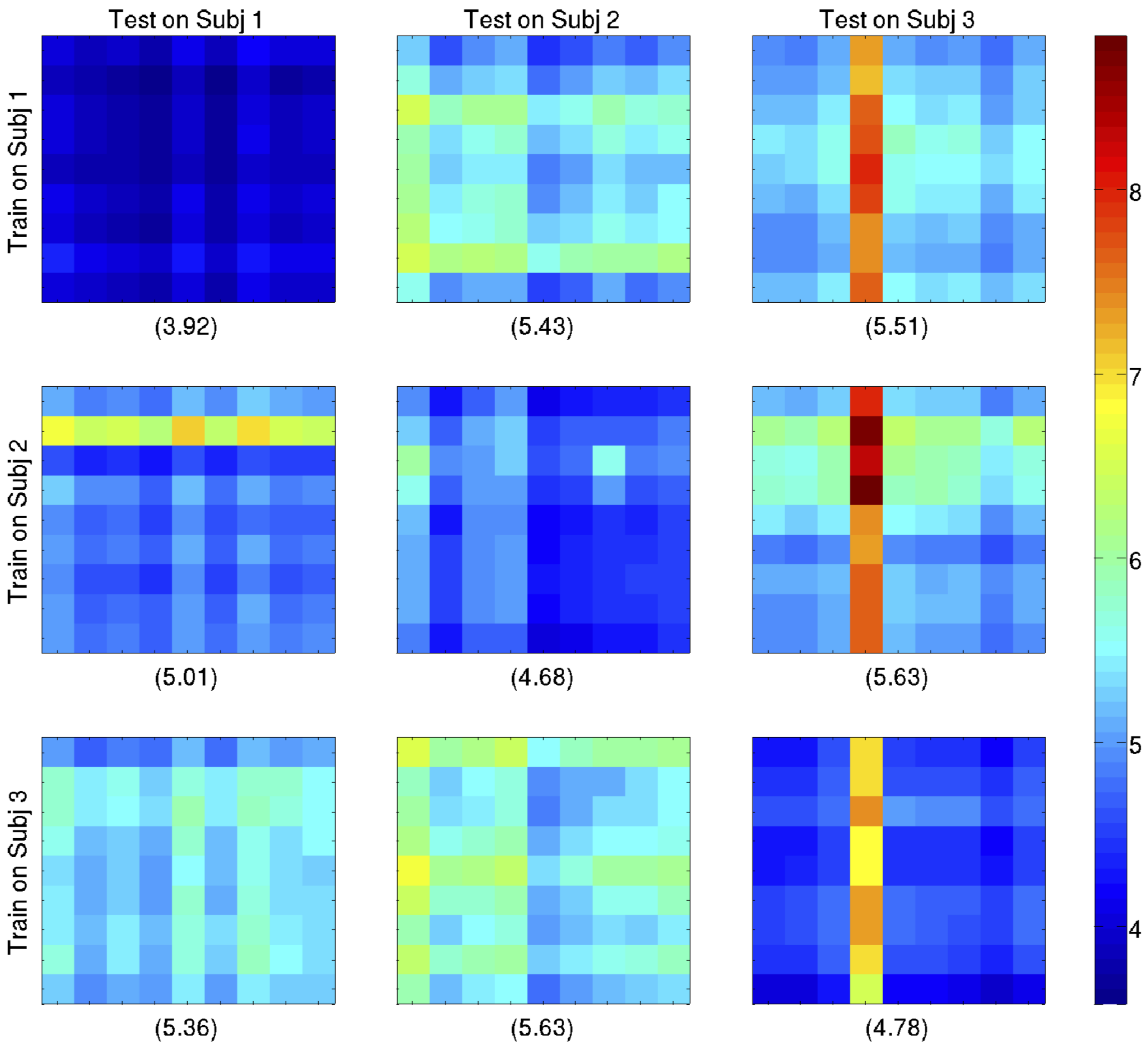

Supplementary Figure 8. The same as Figure 7 while training and testing segments are from three different subjects.

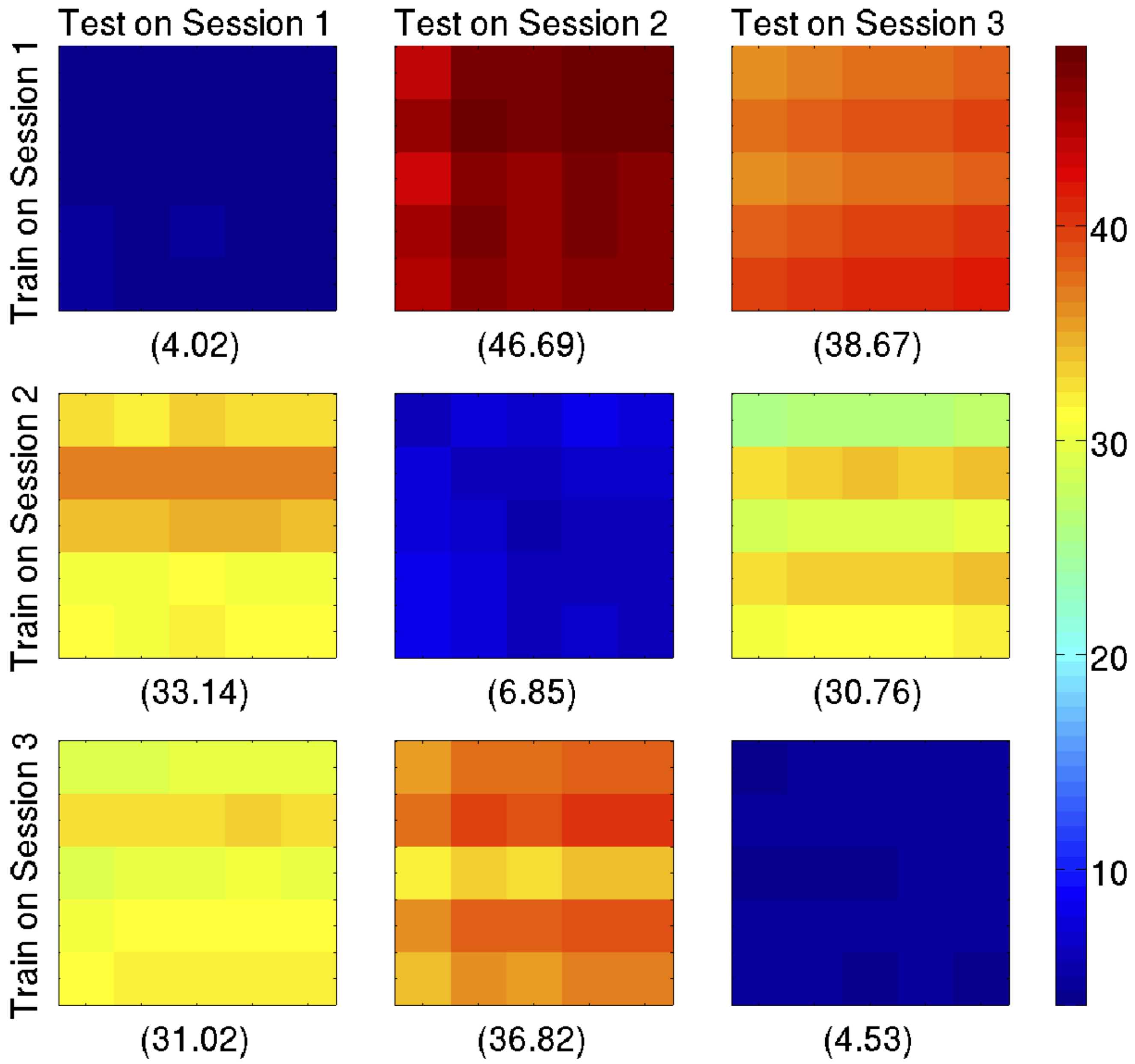

Supplementary Figure 9. BCG estimation errors ($ave\ nRMSE$) in percentage (%) for models whose subset of channels were learned from one training segment and applied to another testing segment of the same subject when inference matrix was also from the training segment.

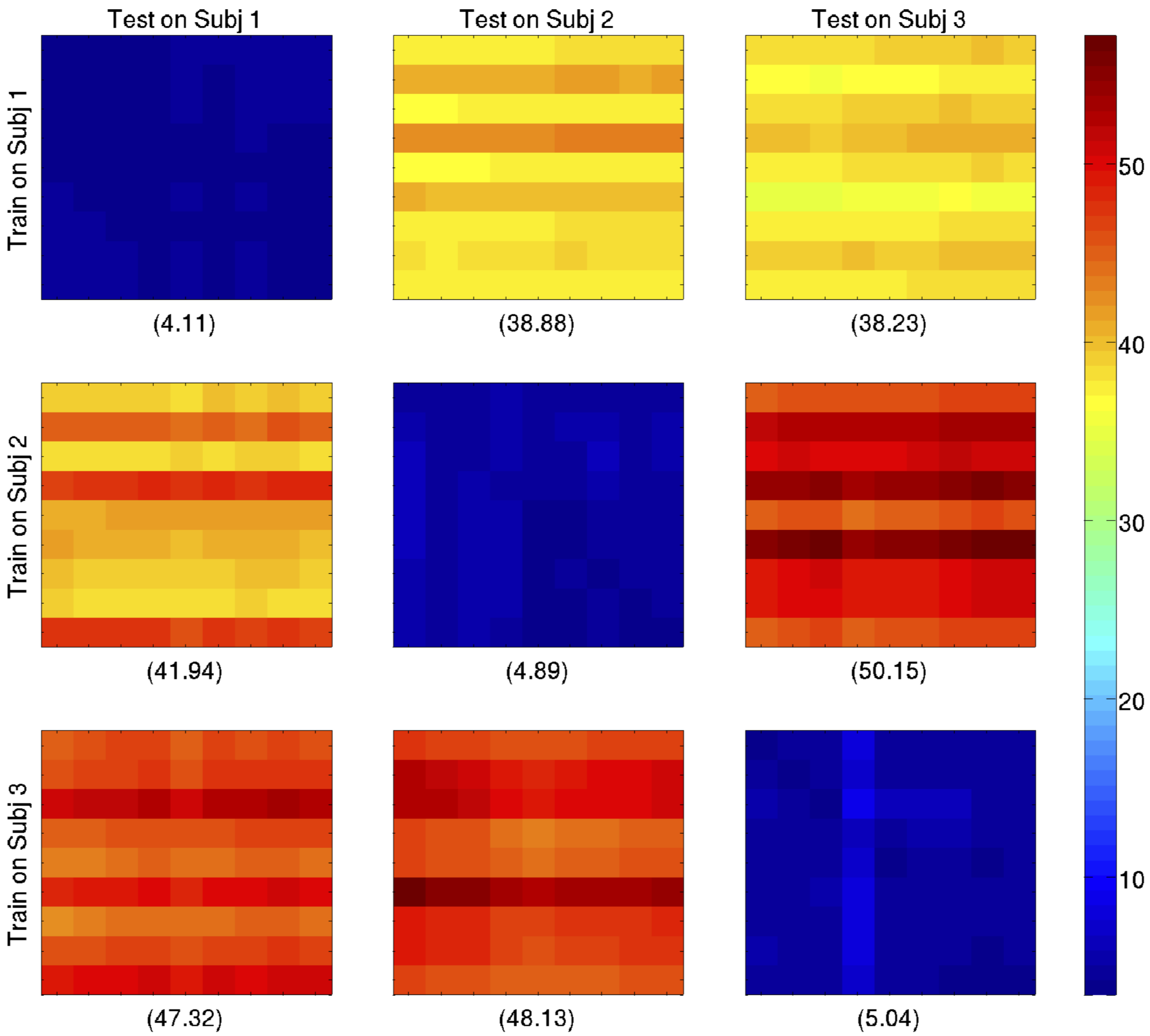

Supplementary Figure 10. The same as Figure 9 while training and testing segments are from four different subjects.

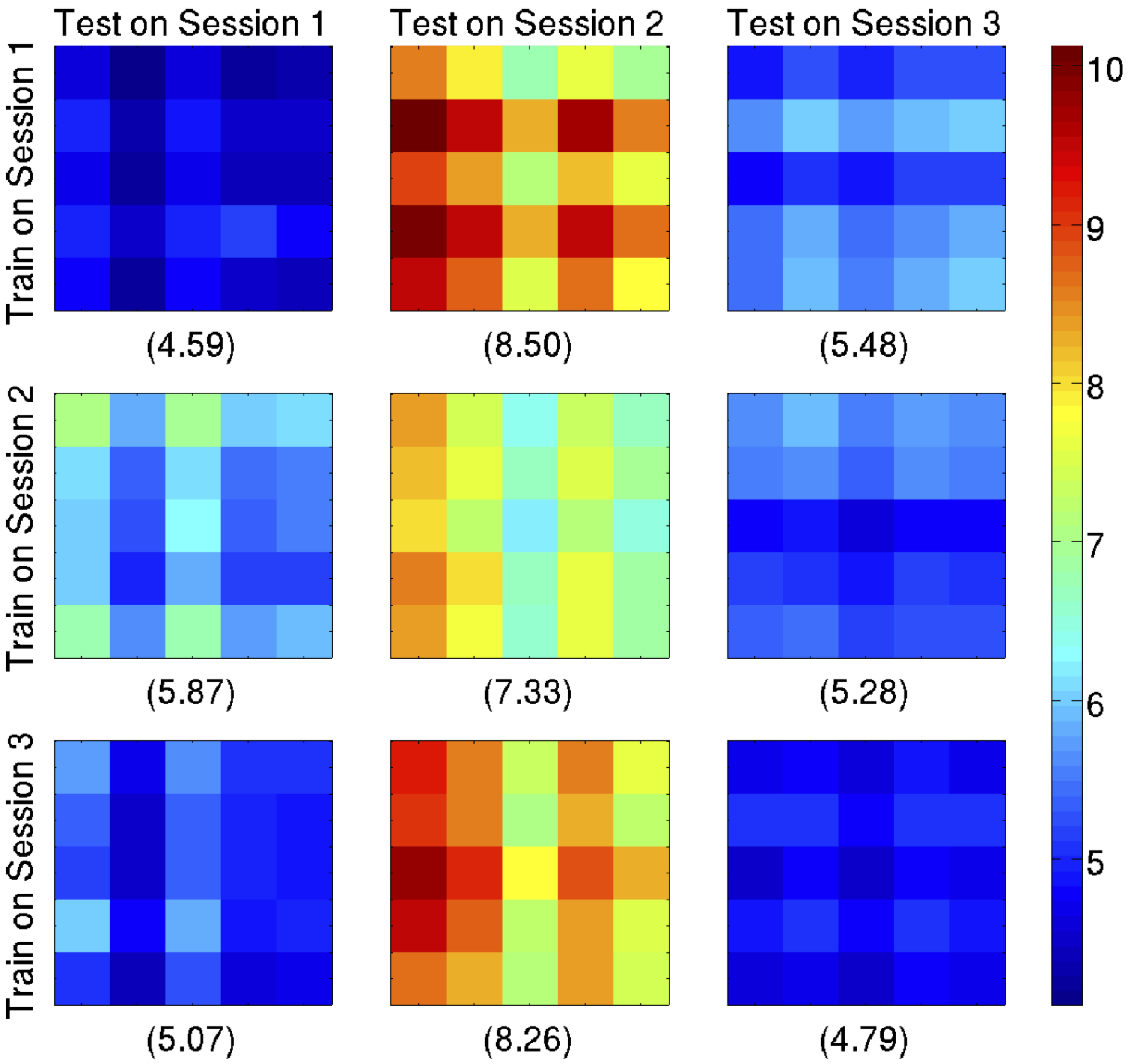

Supplementary Figure 11. BCG estimation errors ($ave\ nRMSE$) in percentage (%) for models whose subset of channels were learned from one training segment and applied to another testing segment of the same subject when corresponding inference matrix was updated with our inference matrix recalculation method.

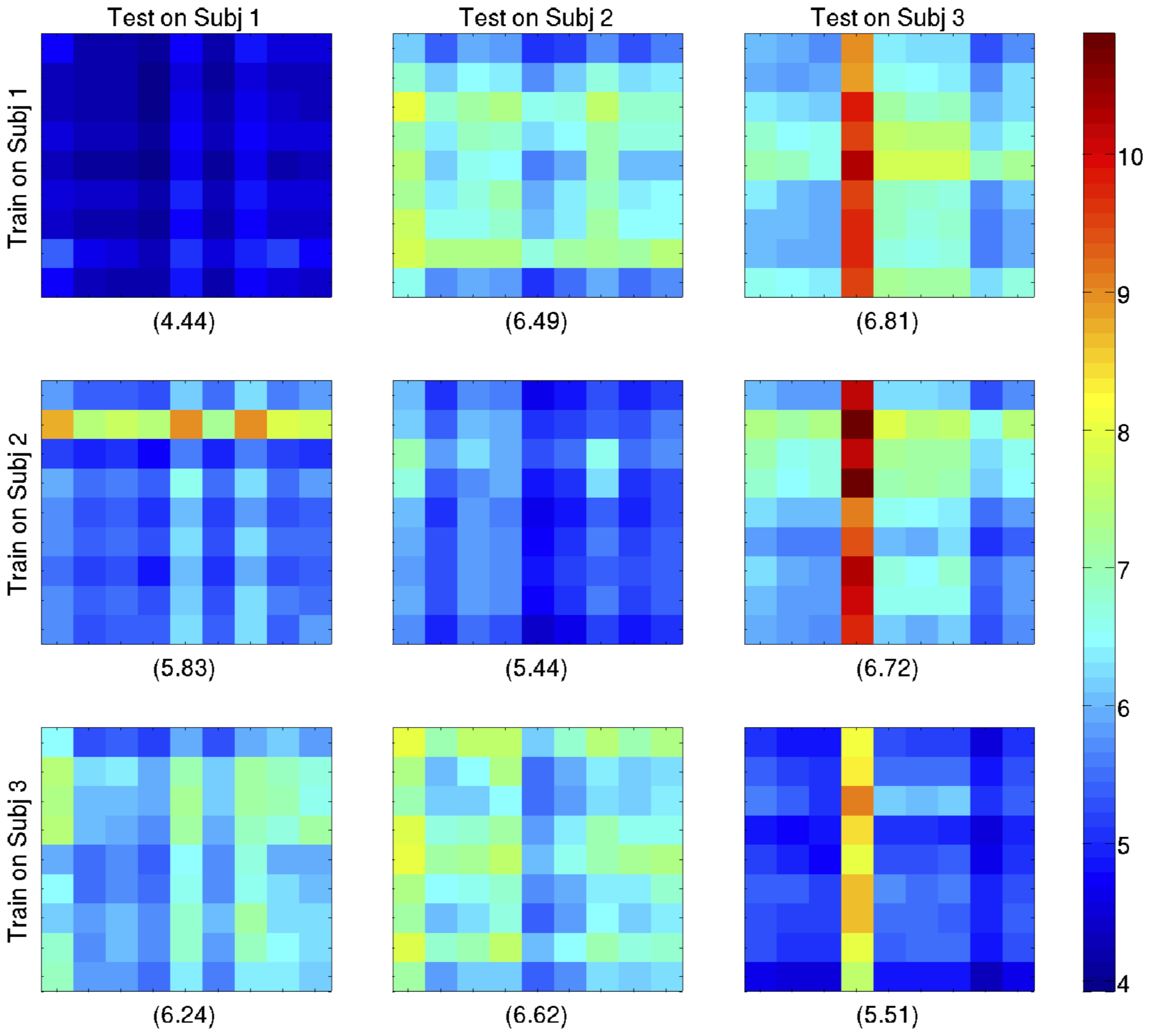

Supplementary Figure 12. The same as Figure 11 while training and testing segments are from four different subjects.

REFERENCES

Allen, P. J., Josephs, O., and Turner, R. (2000), A method for removing imaging artifact from continuous eeg recorded during functional mri, *NeuroImage*, 12, 2, 230–9

Niazy, R. K., Beckmann, C. F., Iannetti, G. D., Brady, J. M., and Smith, S. M. (2005), Removal of fmri environment artifacts from eeg data using optimal basis sets, *NeuroImage*, 28, 3, 720–737
